# Evolutionary significance and diversification of the phosphoglucose isomerase genes in vertebrates

**DOI:** 10.1186/s13104-015-1683-x

**Published:** 2015-12-18

**Authors:** Mbaye Tine

**Affiliations:** Molecular Zoology Laboratory, Department of Zoology, University of Johannesburg, Auckland Park, 2006 South Africa; Genome Centre Cologne at MPI for Plant Breeding Research, 22 Carl-von-Linné-Weg 10, 50829 Cologne, Germany

**Keywords:** Duplication, Divergence, Function, Fishes, Synteny

## Abstract

**Background:**

Phosphoglucose isomerase (PGI) genes are important multifunctional proteins whose evolution has, until now, not been well elucidated because of the limited number of completely sequenced genomes. Although the multifunctionality of this gene family has been considered as an original and innate characteristic, PGI genes may have acquired novel functions through changes in coding sequences and exon/intron structure, which are known to lead to functional divergence after gene duplication. A whole-genome comparative approach was used to estimate the rates of molecular evolution of this protein family.

**Results:**

The results confirm the presence of two isoforms in teleost fishes and only one variant in all other vertebrates. Phylogenetic reconstructions grouped the PGI genes into five main groups: lungfishes/coelacanth/cartilaginous fishes, teleost fishes, amphibians, reptiles/birds and mammals, with the teleost group being subdivided into two subclades comprising PGI1 and PGI2. This PGI partitioning into groups is consistent with the synteny and molecular evolution results based on the estimation of the ratios of nonsynonymous to synonymous changes (Ka/Ks) and divergence rates between both PGI paralogs and orthologs. Teleost PGI2 shares more similarity with the variant found in all other vertebrates, suggesting that it has less evolved than PGI1 relative to the PGI of common vertebrate ancestor.

**Conclusions:**

The diversification of PGI genes into PGI1 and PGI2 is consistent with a teleost-specific duplication before the radiation of this lineage, and after its split from the other infraclasses of ray-finned fishes. The low average Ka/Ks ratios within teleost and mammalian lineages suggest that both PGI1 and PGI2 are functionally constrained by purifying selection and may, therefore, have the same functions. By contrast, the high average Ka/Ks ratios and divergence rates within reptiles and birds indicate that PGI may be involved in different functions. The synteny analyses show that the genomic region harbouring PGI genes has independently undergone genomic rearrangements in mammals versus the reptile/bird lineage in particular, which may have contributed to the actual functional diversification of this gene family.

**Electronic supplementary material:**

The online version of this article (doi:10.1186/s13104-015-1683-x) contains supplementary material, which is available to authorized users.

## Background

The early vertebrate evolution has been characterised by a number of whole genome duplications (WGD) [1–4]. Two rounds of WGD namely 1R and 2R have occurred in vertebrate common ancestor [5–8]. An additional WGD, relatively more recent, has specifically occurred in teleost fish common ancestor [9, 10]. Whole genome, fragment or single gene duplication is amongst the central evolutionary mechanisms that create genomic innovation through the generation of new genetic variants that confer to organisms a better adaptive capacity to their environment [11–14]. Gene duplication produces two paralogues, one of which is free of selective constraints and may accumulate deleterious mutations and eventually becomes a pseudogene [15, 16]. Such non-functional duplicates can be retained in the genome but remain unexpressed and dysfunctional, although more recent studies on gene duplication suggest that pseudogenes might serve some functions [17–19]. The pseudogenes are deleted from the genome or become very divergent from the parental gene so that they are no longer recognisable as such [16, 20]. In rare cases, both paralogues are maintained active because they differ in their functional aspects [16, 21, 22]. The evolutionary scenario under which the duplicate adopts a part of the function of the parental gene is known as subfunctionalisation [15, 16, 23], while neofunctionalisation is applied where one duplicate evolves a different and novel function [23–25]. The neofunctionalisation requires important genetic changes in the key amino acid positions that are central determinants of the function of the protein [23, 26]. Changes in nucleotides and/or amino acid composition are not the only genetic changes that can lead to functional divergence after gene duplication [27, 28]. Genomic rearrangements including spontaneous genomic deletions may occur and cause a complete loss of some introns, which may lead to changes in exon length and nucleotide compositions [29, 30]. The fusion of remaining adjacent exons following intron loss may create new variants that conserve a part of the parental function or involve a completely different novel function [31, 32].

The observations of differential conservation or loss of duplicates has led to the question of why some variants are conserved in some species and lost in closely related species [33, 34]. This has also raised the question whether the absence of certain duplicates in genomes implies the loss of specific functions or whether the function that they were fulfilling is accomplished by other members of the same gene network or physiological pathways [24]. The relative frequency of duplicate loss or conservation varies considerably among organisms, even between species within the same lineage [35, 36]. An exhaustive analyses of aquaporin and claudin genes in teleost fishes revealed significant differences in the number of paralogs of these gene families between species [37–39]. Other examples of differential duplicate conservation or loss have been reported for many gene families in euteleosts [40, 41]. It cannot be ruled out that the failure to identify duplicates in teleost genomes was an artefact of sequence incompleteness due to low sequencing coverage for these genomes [42, 43]. The availability of many genomes distributed over different kingdoms now allows to address such questions (at a large scale).

The phosphoglucose isomerage (PGI) is amongst the genes that have retained both paralogs after teleost-specific WGD [44–47]. The PGI protein gene has been identified as a key enzyme of the glycolysis pathway, where it insures the inter conversion between d-glucose-phosphate and d-fructose-6-phosphate [44, 48]. PGI is also involved in other functions, including thermal adaptation, differentiation and mediation of maturation inducer activity, which might result from secondary effects of the basic function [44, 48, 49]. The protein structure of PGI genes in relation to their genomic evolution has been investigated by a few studies using a limited number of taxa [45–47] that did not cover the whole vertebrates. It has been thus demonstrated that the electric charges of teleost PGI1 and PGI2 have significantly diverged, which was interpreted as a sub-functionalisation indicating that the two PGI paralogs have evolved to have different functions after duplication [47]. The same authors tried to infer the origin of this sub-functionalisation by applying an evolutionary model to identity sign of positive selection after PGI duplication. However, the results were not clear, indicating that the evolutionary processes that has led to the functional divergence of PGI1 and PGI2 are still not completely understood and need further evaluation. Although large divergence rate between duplicates could be the result of positive selection on novel function, relaxation of selective constraints, and even loss of function, the levels of sequence divergence can provide information on the process of neofunctionalisation among duplicates [50, 51]. The divergence rates of protein sequences are expected to be higher in duplicates that have evolved novel functions compared to those that did not undergone neofunctionalisation. Therefore, an estimation of the ratios of non-synonymous to synonymous changes and divergence rates between PGI1 and PGI2 paralogs and orthologs among and within lineages may help to infer the evolutionary origin and support previous findings on the functional divergence of teleost PGI1 and PGI2.

The objective of the present study was to infer and retrace the evolutionary history of the PGI genes in vertebrates by combining similarity, phylogenetic and conserved synteny analyses. Another objective of this study was to estimate the divergence times between PGI pairwise paralogs and orthologs among and within lineages in order to infer the origin and confirm the functional diversification of this gene family that has been previously reported [46, 47]. The results show that, in addition to the functional divergence resulting from amino acid changes, complex genomic rearrangements including inversion, intron gain and intron deletion have also affected the region harbouring PGI genes after duplication, which has probably led to their actual functional diversification.

## Results

### Synteny analyses

**Fig. 1:**
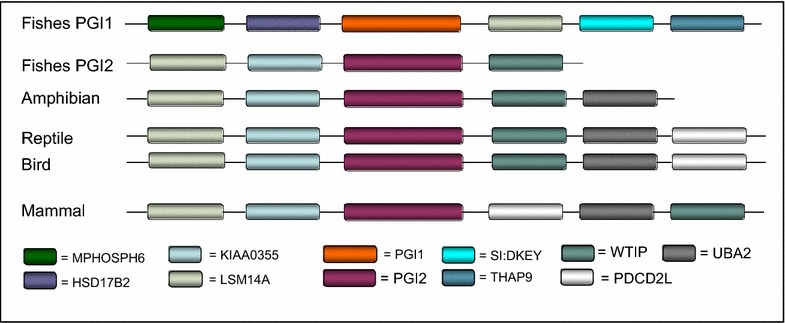
The general organisation of genes surrounding PGI genes

**Fig. 2:**
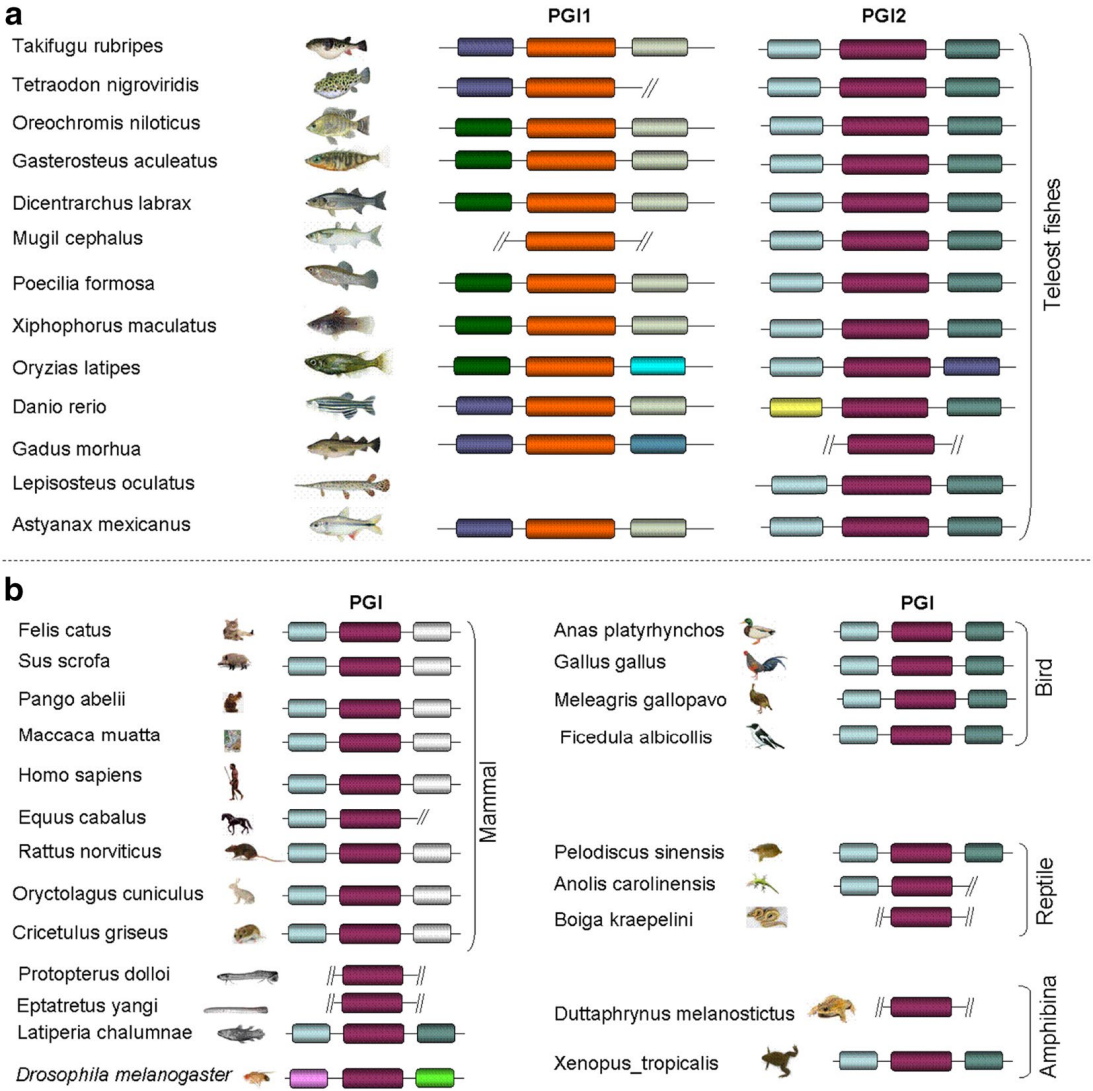
Conserved synteny of PGI and their upstream and downstream flanking genes: teleost fishes (**a**), Amphibians, reptiles, birds, mammals, hagfishes, lamprey and the coelacanth (**b**). The different colors surrounding PGI isoforms indicate the different flanking upstream and downstream flanking genes. The names of different flanking genes are indicated in Table 1

The genomic location of PGI genes identified in all analysed species as well as their flanking genes is shown in Table 1. Two PGI genes were found in all teleost fishes. The similarity and synteny analyses resealed that these two isoforms correspond to two variants that have been previously characterised in *Danio rerio*. These two isoforms are referred as PGI1 and PGI2 in this study. Only one PGI isoform was found the in the holostei, the spotted gar *Lepisosteus oculatus* (Table 1). The first and/or second flanking gene(s) was lost in some of the species. Therefore, the two first adjacent upstream flanking genes: *MPHOSPH6*: (*UFG1)* and *HSD17B2 (UFG2)* of PGI1 isoform were presented. Likewise, the three first adjacent downstream flanking genes: *LSM14A* (*DFG1), SI:DKEY (DFG2)* and, *THAP9 (DFG3)* are also shown in Table 1. Similarly, the two first upstream flanking genes (*KIAA0355* and *LSM14A*) and downstream adjacent flanking genes (*WTIP* and *HSD17B2*) of PGI2 are also indicated in Table 1. PGI1 was located on corresponding chromosomes 25 and 6 in *D. rerio* and *Oryzias latipes* genomes [52], respectively, while PGI2 was located on corresponding *Dicentrarchus labrax* (LG5) and *Gasterosteus aculeatus* (GroupII) chromosomes [53]. More importantly, *O. latipes* PGI1 and PGI2 are located on two distinct chromosomes (6 and 3) that share a high degree of synteny [54], suggesting that they may have resulted from a duplication of the same genomic region. Also, only one PGI gene was identified in amphibians, reptiles, birds and mammals. The two PGI isoforms identified in fishes are located on different chromosomes. The order of PGIs and their flanking genes is shown in Fig. 1 and Table 1. This order varied between PGI isoforms, between lineages and even within lineages. The PGI1 gene in most teleost species (including the Atlantic cod, *Gadus morhua*, the zebrafish, *D. rerio*, *Astyanax mexicanus*, the two pufferfish *Tetraodon nigorviridis* and *Takifugu rubripes*) is flanked upstream and downstream by the hydroxysteroid (17-beta) dehydrogenase 2 (*HSD17B2*) and *LSM14A* mRNA processing body assembly factor a (*LSM14AA*) genes, respectively (Fig. 2a). By contrast, in some species including the stickleback, *G. aculeatus*, medaka, *O. latipes*, the tilapia, *Oreochromis niloticus*, Amazon molly, *Poecilia Formosa* and the platyfish, *Xiphophorus maculatus*, the PGI1 gene is flanked upstream by the M-phase phosphoprotein 6 (*MPHOSPH6*) gene (Fig. 2a), which is the upstream gene of *HSD17B2* gene. The second teleost isoform, PGI2 is flanked upstream by the *KIAA0355* gene whereas its downstream flanking gene is Wilms tumor 1 interacting protein (*WTIP*) (Fig. 2a). Only in *O. latipes* is PGI2 flanked downstream by a different gene*, HSD17B2* (Fig. 2a) i.e. the upstream flanking gene of PGI1.

**Table 1:** Gene and protein ID and genomic location of PGI loci identified in the different species

Species scientific name	Species common name	*UFG2*	*UFG1*	*PGI locus*	*DFG1*	*DFG2*	*DFG3*	Gene ID	Protein ID	Genomic location	Genomic position	Strand
Fishes		*MPHOSPH6*	*HSD17B2*	*PGI1*	*LSM14A*	*SI:DKEY*	*THAP9*					
*Astyanax mexicanus*	Mexican tetra		*HSD17B2*	*PGI1*	*LSM14A*			ENSAMXT00000018221	ENSAMXP00000018221	Scaffold KB871579.1	7,816,843–7,827,707	Forward
*Danio rerio*	Zebrafish		*HSD17B2*	*PGI1*	*LSM14A*			ENSDART00000022437	ENSDARP00000009909	Chromosome 25	37,001,059–37,026,441	Forward
*Gasterosteus aculeatus*	Three-spined stickleback	*MPHOSPH6*		*PGI1*	*LSM14A*			ENSGACT00000010328	ENSGACP00000010306	GroupXIX	8,855,280–8,863,611	Forward
*Oreochromis niloticus*	Nile tilapia	*MPHOSPH6*		*PGI1*	*LSM14A*			ENSONIT00000022998	ENSONIP00000022978	Scaffold GL831424.1	532,106–539,134	Forward
*Oryzias latipes*	Medaka	*MPHOSPH6*		*PGI1*		SI:DKEY		ENSORLT00000000310	ENSORLP00000000310	Chromosome 6	336,560–345,710	Reverse
*Poecilia formosa*	Amazon molly	*MPHOSPH6*		*PGI1*	*LSM14A*			ENSPFOT00000000722	ENSPFOP00000000721	Scaffold KI520292.1	32,646–43,864	Forward
*Dicentrarchus labrax*	European seabass	*MPHOSPH6*		*PGI1*	*LSM14A*			DLAgn_00260200	DLAgn_00260200	UN:85995004-86018309		
*Takifugu rubripes*	Japanese pufferfish		*HSD17B2*	*PGI1*	*LSM14A*			ENSTRUT00000006700	ENSTRUP00000006657	scaffold_526	29,664–34,652	Reverse
*Tetraodon nigroviridis*	Spotted green pufferfish		*HSD17B2*	*PGI1*	??			ENSTNIT00000023294	ENSTNIP00000023052	Chromosome Un_random	103,153,150–103,157,885	Forward
*Gadus morhua*	Atlantic cod		*HSD17B2*	*PGI1*			THAP9	ENSGMOT00000006028	ENSGMOP00000005855	Scaffold_515	95,640–108,132	Forward
*Mugil cephalus*	Striped Mullet		??	*PGI1*	??							
*Xiphophorus maculatus*	Southern platyfish	*MPHOSPH6*		*PGI1*	*LSM14A*			ENSXMAT00000007135	NSXMAP00000007127	Scaffold JH556666.1	4,515,304–4,527,053	Reverse

Fishes		*KIAA0355*	*LSM14A*	*PGI*	*WTIP*	*HSD17B2*						
*Astyanax mexicanus*		*KIAA0355*		*PGI2*	*WTIP*			ENSAMXT00000021636	ENSAMXP00000021636	Scaffold KB882149.1	2,238,557–2,258,490	Forward
*Danio rerio*				*PGI2*	*WTIP*			ENSDART00000020914	ENSDARP00000014578	Chromosome 13	51,999,268–52,020,986	Forward
*Gadus morhua*			??	*PGI2*	*??*			ENSGMOT00000016031	ENSGMOP00000015632	Scaffold_3436	42,484–54,532	Forward
*Gasterosteus aculeatus*		*KIAA0355*		*PGI2*	*WTIP*			ENSGACT00000019782	ENSGACP00000019744	GroupII	5,627,138–5,632,419	Forward
*Oreochromis niloticus*		*KIAA0356*		*PGI2*	*WTIP*			ENSONIT00000003600	ENSONIP00000003599	Scaffold GL831133.1	4,705,311–4,715,207	Reverse
*Oryzias latipes*		*KIAA0355*		*PGI2*		*HSD17B2*		ENSORLT00000017655	ENSORLP00000017654	Chromosome 3	29,723,585–29,732,093	Reverse
*Poecilia_formosa*		*KIAA0355*		*PGI2*	*WTIP*			ENSPFOT00000015130	ENSPFOP00000015108	Scaffold KI519656.1	685,683–695,888	Forward
*Dicentrarchus labrax*		*lKIAA0356*		*PGI2*	*WTIP*			DLAgn_00155520	DLAgn_00155520	LG5	5,056,240–5,064,239	
*Takifugu rubripes*		*KIAA0355*		*PGI2*	*WTIP*			ENSTRUT00000040726	ENSTRUP00000040584	scaffold_91	917,007–924,134	Forward
*Tetraodon nigroviridis*		*KIAA0355*		*PGI2*	*WTIP*			ENSTNIT00000013624	ENSTNIP00000013430	Chromosome Un_random	33,376,190–33,381,003	Reverse
*Xiphophorus maculatus*		*KIAA0355*		*PGI2*	*WTIP*			ENSXMAT00000016551	ENSXMAP00000016527	Scaffold JH556668.1	1,362,881–1,372,790	Reverse
*Lepisosteus oculatus*	Spotted gar	*KIAA0355*		*PGI*	*WTIP*			ENSLOCT00000002438	ENSLOCP00000002433	Chromosome LG23	2,922,532–2,940,796	Forward

Mammalians		*LSM14A*	*KIAA035*	*PGI*	*WTIP*	*PDCD2L*	*UBA2*					
*Equus caballus*	Horse		*KIAA0355*	*PGI*				ENSECAT00000020155	ENSECAP00000016531	Chromosome 10	5,434,436–5,460,230	Forward
*Felis catus*	Cat		*KIAA0355*	*PGI*			PDCD2L	ENSFCAT00000005279		Chromosome E2	18,208,924–18,234,333	Reverse
*Homo sapiens*	Human		*KIAA0355*	*PGI*			*PDCD2L*	ENST00000415930	ENSP00000405573	Chromosome 19	34,364,740–34,402,156	Forward
*Macaca mulatta*	Rhesus macaque		*KIAA0355*	*PGI*			PDCD2L	ENSMMUT00000008231	ENSMMUP00000007737	Chromosome 19	40,926,355-40,964,914	Forward
*Oryctolagus cuniculus*	Rabbit		*KIAA0355*	*PGI*			PDCD2L	ENSOCUT00000001455	ENSOCUP00000001250	Chromosome 5	4,169,177–4,197,347	Forward
*Pongo abelii*	Sumatran orangutan		*KIAA0355*	*PGI*			PDCD2L	ENSPPYT00000011442	ENSPPYP00000011012	Chromosome 19	35,031,028–35,068,667	Forward
*Sus scrofa*	Eurasian wild pig		*KIAA0355*	*PGI*			PDCD2L	ENSSSCT00000003175	ENSSSCP00000003094	Chromosome 6	39,517,086–39,547,942	Reverse
*Rattus norvegicus*	Norwegian rat		*KIAA0355*	*PGI*			PDCD2L	ENSRNOT00000032613	ENSRNOP00000029515	Chromosome 1	91,207,014–91,234,890	Reverse
*Mus musculus*	House mouse			*PGI*				ENSMUST00000038027	ENSMUSP00000049355	Chromosome 7	34,202,122–34,230,281	Reverse

Avians/reptiles/amphibians		*LSM14A*	*KIAA0355*	*PGI*	*WTIP*	*UBA2*	PDCD2L					
*Ficedula albicollis*	Collared flycatcher		*KIAA0355*	*PGI*	*WTIP*			ENSFALT00000008115	ENSFALP00000008082	Scaffold JH603268.1	2,075,371–2,103,822	Reverse
*Gallus gallus*	Chicken		*KIAA0355*	*PGI*	*WTIP*			ENSGALT00000007948	ENSGALP00000007934	Chromosome 11	10,450,875–10,471,700	Forward
*Meleagris gallopavo*	Common turkey		*KIAA0355*	*PGI*	*WTIP*			ENSMGAT00000006817	ENSMGAP00000006071	Chromosome 13	10,920,111–10,933,784	Forward
*Anas platyrhynchos*	Mallard		*KIAA0355*	*PGI*	*WTIP*			ENSAPLT00000010618	ENSAPLP00000009916	Scaffold KB743139.1	1,702,356–1,722,763	Forward
*Pelodiscus sinensis*	Chinese softshell		*KIAA0355*	*PGI*	*WTIP*			ENSPSIT00000012892	ENSPSIP00000012831	Scaffold JH210905.1	2,814,958–2,841,275	Reverse
*Boiga kraepilini*	Widespread snake		??	*PGI*	??							
*Duttaphrynus melanostictus*	Asian common toad		??	*PGI*	??			tr|Q8QFU6|Q8QFU6_DUTME				
*Xenopus tropicalis*	Western Clawed Frog		*KIAA0355*	*PGI*	*WTIP*			ENSXETT00000005036	ENSXETP00000005036	Scaffold GL172806.1	377,293-398,724	Reverse
*Anolis carolinensis*	Carolina anole		*KIAA0355*	*PGI*	??			ENSACAT00000001365	ENSACAP00000001332	Scaffold GL343773.1	142,574–185,909	Forward

Other lineages			*PGI*									
*Protopterus dolloi*	African lungfishes	??	*PGI*	??				gi|44965330|gb|AY389912.1	gi|44965331|gb|AAS49536.1|			
*Petromyzon marinus*	Sea Lamprey		*PGI*					ENSPMAT00000004427	ENSPMAP00000004410	Scaffold GL481891	3,996–20,619	
*Drosophila melanogaster*	Fruit fly		*PGI*					FBtr0088679	FBpp0087760	Chromosome 2R	4,798,407–4,801,631	Reverse
*Latimeria chalumnae*	Coelacanth	*KIAA0355*	*PGI*	*WTIP*				ENSLACT00000000911		Scaffold JH128875.1	5,026–58,037	Reverse

*UFG1*, upstream flanking gene 1; *UFG2*, upstream flanking gene 2*, DFG1*, downstream flanking gene 1; *DFG2*, downstream flanking gene 2; *DFG3*, downstream flanking gene 3

The PGI gene identified in amphibians (*Duttaphrynus melanostictus* and *Xenopus tropicalis*), reptiles (*Pelodiscus sinensis, Anolis carolinensis*) and birds (*Ficedula albicollis, Anas platyrhynchos* and *Meleagris gallopavo*) is flanked upstream by *KIAA0355* whereas the downstream flanking gene is *WTIP* (Fig. 2b), which is also the downstream flanking gene of teleost PGI2 genes. The mammalian PGI gene has the same upstream flanking gene (*KIAA0355*) as amphibians, reptiles and birds but their downstream flanking gene is the programmed cell death 2-like gene (*PDCD2L*) in *Mus musculus*, *Rattus norvegicus*, *Pongo abelii*, *Macaca mulatta*, *Homo sapiens* (Fig. 2b) and *ENSECAT00000021114* in *Equus caballus*, uncharacterized protein *ECO:0000313* in *Sus scrofa*, *ENSOCUT00000001459* in *Oryctolagus cuniculus* and uncharacterized protein, *ECO:0000313*, in *Felis catus*. The similarity search in Ensembl using BLAT showed very high similarity in these uncharacterised proteins to *PDCD2L*, suggesting that all mammals analysed in this study have the same downstream gene, *PDCD2L*, a locus located just before the ubiquitin-like modifier activating enzyme 2 (*UBA2*), which flanks the *WTIP* at its upstream part. In other words, there are two genes between mammalian PGI gene and the *WTIP* gene, which flanks the PGI of vertebrates including teleost PGI2. Based on these syntenic analyses, the PGI gene in amphibians, reptiles, birds and mammals present more similarities with the PGI2 of fishes, and can therefore be qualified as PGI2, as annotated in the GenBank and Ensembl database for most of the species.

In the lamprey, *Petromyzon marinus,* only one PGI gene was found and its upstream and downstream flanking genes were not identified (Fig. 2b). This PGI gene was the only gene found on the scaffold where it is located. Similarity search using fishes flanking genes did not allow identifying the bordering genes in other scaffolds of the genome. The failure to identify the lamprey PGI flanking genes could be due to the quality of the assembly, which is problematic in this species. In the coelacanth, *Latimeria chalumnae*, only one PGI gene was identified, which is located on the same scaffold as its downstream flanking gene, *WTIP*. Its upstream flanking gene was found at the extremity of a different scaffold (Scaffold *JH127461.1*). The similarity and syntenic analyses indicated that this PGI is more similar to the PGI2 of the other species than to the PGI1 gene. The BLAST search using teleost fish PGI1 gene and its upstream and downstream genes did not reveal the presence of another PGI gene in coelacanth and lamprey, suggesting that these two species have only one PGI isoform. The similarity search identified only one PGI isoform in the elephant shark, *Callorhinchus milii,* and the lancelet *Branchiostoma floridae,* and their flanking genes were not successfully identified.

To better understand the origin of PGI gene, I searched for its presence in invertebrate genomes. Similarity search identified only one gene in the Fruitfly, *Drosophila melanogaster*, flanked upstream and downstream by FBtr0088650 and FBgn0002552, respectively (Fig. 2b). The similarity search indicates that these two genes are different from the upstream and downstream flanking genes of PGI genes found in the other species. The *D. melanogaster* (DM) PGI gene has a smaller number of exons (5 exons only) compared to the PGI genes of all vertebrate species (Additional file1: Table S1).

### Structure of PGI genes

Most of the PGI genes identified in this study comprise 18 exons interrupted by 17 introns. The PGI1 gene of *L. oculatus* (LO) and *S. scrofa* (SS) has an additional exon (Additional file 1: Table S1). In *T. nigroviridis* (TN), both PGI isoforms have 17 exons. In *A. mexicanus* (AM), PGI1 has 17 exons and PGI2 18 exons whereas in *G. morhua* (GM), PGI1 consists of 18 exons and PGI2 17 exons. *P. sinensis* (PS) also have 17 exons and 16 introns (Additional file 1: Table S1). The smaller number of exon in vertebrates was found in *P. marinus* (MP) and *M. gallopavo* (MG), 11 and 15 exons, respectively (Additional file 1: Table S1). The multiple alignment of DM PGI with other vertebrate PGI did not show differences in protein sequence lengths. By contrast, the multiple alignment of nucleotide sequences of PM PGI with *D. rerio* PGI1 and PGI2 shows that the upstream and downstream exons were lost in this species. Similar results were observed for MG whose the amino acid sequence alignment with GG shows that the first part the sequence is missing, suggesting a deletion the first exons.

In fishes, the largest exon for both PGI isoforms is exon 18 with a maximum length of 2227 bp for *P. formosa* (PF) PGI1 (Additional file 1: Table S1). In the pufferfish, *T. rubripes* (TR) and *T. nigroviridis*, the largest exons were respectively exons 12 and 11 with 153 bp each. in *G. morhua* (GM), the largest exon for both PGI isoforms was exon 12 with a total length of 153 bp (Additional file 1: Table S1). In all fish species, the smallest exon was exon 11 with 44 bp except for *T. nigroviridis* where the smallest exon was exon 13 with a total length of 22 bp. In *M. gallopavo*, the smallest exon is comprised of 44 bp but it was exon 8 instead of exon 11 like in the other species (Additional file 1: Table S1). Likewise, the shortest exon for the other eukaryote PGI genes was exon 11 with 44 bp except in *L. oculatus* PGI, whose exon 11 was of 22 bp in length. In *P. sinensis*, the shortest exon was exon 1, which count 21 bp. In *D. melanogaster* the largest exon was exon 1 whereas the shortest was exon 3. The exon 11 was the most conserved between PGI isoforms and between species in term of length. Other exons such as 5, 6, 7, 8, 9 and 11 were also very conserved in term of length between PGI isoforms but also between species. (Additional file 1: Table S1) The upstream and downstream exons seemed to be more variable in term of size for all PGI genes and in all species. The largest and smallest intron was not the same for PGI paralogs and were also variable between PGI orthologs, i.e. between species (Additional file 1: Table S2). For example in *G. aculeatus*, the largest introns were introns 3–4 (1857 bp) for PGI1 and intron 9–10 (525 bp) for PGI2 whereas the smallest introns were respectively intron 5–6 (75 bp) and 4–5 (84 bp) (Additional file 1: Table S2). In the pufferfish, the largest PGI1intron was intron 1–2 (1079 bp) in *T. rubripes* and 14–15 (2101 bp) for *T. nigroviridis* (Additional file 1: Table S2). The smallest intron for PGI1 in both species was the same, intron 12–13 but the length was very different, 76 bp for *T. rubripes* versus 4 bp for *T. nigroviridis*.

### Principal component analysis

**Fig. 3:**
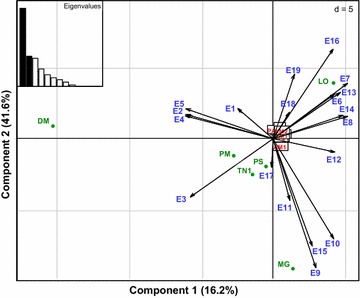
Principal component analysis map showing the distribution of PGI genes in different vertebrates according to their exon length. The name given to the PGI gene on the PCA plot represents the initials of the scientific name of each species, followed by a number which refer to the isoform. The *blue color* indicates the exon. The *red color* represents all vertebrate PGI clustered together in the same group while the *green* indicates exceptions, i.e. PGI genes isolated from this group

The principal component analysis (PCA) allowed to differentiate the PGI genes into different groups based on the number of exons as well as their length (Fig. 3). All vertebrate PGI genes were clustered in a same group, except *L. oculatus* (LO), *M. gallopavo* (MG), *P. marinus* (PM) and *P. sinensis* (PS) PGI gene, and *T. nigroviridis* PGI1(TN1) which are completely distinct on the PCA plot. The PGI of these species are also not grouped together on the PCA plot (Fig. 3). The position of LO on the PCA plot is determined by the exon E16 whereas that of DM is due to the exons E2, E4 and E5 (Fig. 3). The position of PM and TN1 on the PCA map is essentially determined by E3 while that of PS is explained by E17 (Fig. 3).

**Fig. 4:**
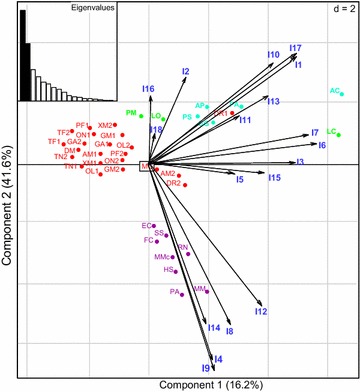
Distribution of PGI genes according to the length of their introns. The name given to PGI on the PCA plot represents the initials of the scientific name of the species, followed by a number which indicates to the isoform. The introns are indicated in *blue*. PGI groups of fishes and mammals are indicated in *red* and *purple*, respectively. The *turquoise* color represents bird/reptile PGI while *bright* color represents the lamprey (*P. marinus*), the spotted gar (*L. oculatus*) and the coelacanth (*L. chalumnae*) PGI that are isolated from the other groups

The repartition of PGI on the PCA plot based on the intron information allowed differentiating vertebrate PGI in three different groups; teleost, reptile/bird and mammal. *D. rerio* PGI1 (DR1) and PGI2 (DR2), and *A. mexicanus* PGI2 (AM2) are the only PGI genes that are isolated from other teleost PGI genes. The coelacanth *L. chalumna*e PGI (LC) and lizard *A. carolinensis* (AC) are very isolated from other PGI genes, but also isolated among themselves (Fig. 4). While the position of LC on the PCA plot is essentially explained by introns I6 and I7, that of AC seems to be determined by more number of introns including I6, I7, I10, I11, I13 and I17 (Fig. 4). Within the mammal lineage only *M. musculus* (MM) and *P. abelii* (PA) PGI genes are isolated in a different sub-group. The mammal group is essentially defined by introns I4, I8, I12, I14 and I19 whereas bird/reptile group is explained by I10, I11, I13 and I17. The position of the teleost group (formed by PGI1 and PGI2 paralogs) on the PCA map is not related to intron length except for DR1, DR2 and AM2, whose the position on the PCA map seems to be related to the intron size (Fig. 4). The lamprey, *P. marinus* (PM) and the spotted gar, *L. oculatus* (LO) PGI genes are isolated but closely related on the PCA map and their repartition their position is essentially explained by I2, I16 and I18 (Fig. 4).

### Phylogenetic analyses

**Fig. 5:**
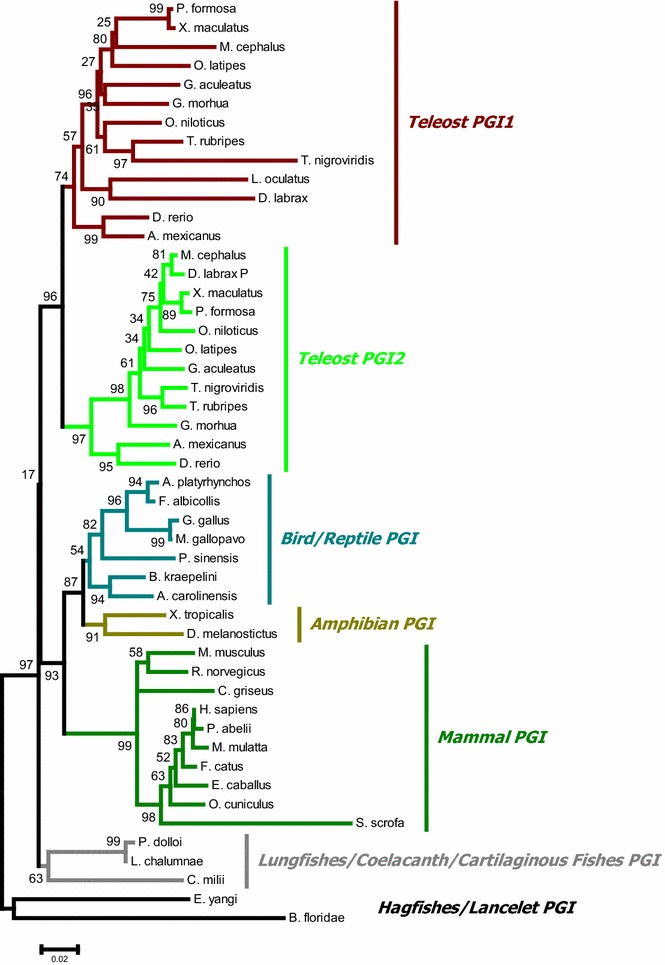
Maximum likelihood phylogenetic tree of vertebrate PGIs. The phylogenetic tree was conducted on 50 protein sequences using MEGA software version 6. The values at the nodes represent the bootstrap values from 1000 replicates. The tree was rooted with hagfishes and lancelet PGI protein sequences

The phylogenetic analysis recovered five main PGI lineage-dependent groups: lungfishes/coelacanth/cartilaginous fishes, teleost fishes, amphibians, reptiles/bird and mammals (Fig. 5). The teleost group is subdivided into two different clades comprising the PGI1 and PGI2 genes, respectively (Fig. 5). All teleost PGI1 genes are grouped in the same clade whereas the PGI2 genes are grouped in a different clade. According to the phylogenetic tree, teleost PGI1 and PGI2 are each other sister group and shows a common origin. The phylogenetic tree do not clearly show which of these two teleost PGI isoforms is more related to the PGI gene of reptiles, birds and mammals which all belong to the same clade, a result expected for WGD that produces two sister groups. The phylogenetic tree including the lamprey PGI sequence is not presented here because it was not well resolved, which may be due to the wrong frameshift found in the annotation of the PGI gene in this species. The within-species phylogenetic results of PGIs in the teleost lineage are not congruent with the taxonomic relationships between species. For example *G. aculeatus* PGI1 is closely related to *G. morhua* PGI1 whereas its PGI2 is more related to PGI2 of other species such as *O. latipes*, *T. nigroviridis* or *T. rubripes*. Likewise, *M. cephalus* PGI1 is closely related to that of *P. formosa* and *X. maculatus* while its PGI2 showed more similarity with *D. labrax* PGI2. Possible reasons for this discrepancy are different evolutionary constraints that may have been impacted on PGI isoforms in these species after duplication.

### Natural selection

The average Ka/Ks ratio (Table 2) measured between teleost PGI1 and PGI2 paralogs is 0.64. The Ka/Ks ratio measured was higher than 1 in *P. formosa* (1.50), *G. morhua* (2.07) and *D. labrax* (1.74) and below the average in all other teleost species (Table 2). This average is higher than that measured between teleost PGI1 and PGI2 orthologs, which were 0.07 and 0.06, respectively. The Ka/Ks ratio measured between teleost PGI1 and mammal PGI was 1.96 whereas the average between teleost PGI2 and mammal PGI was 1.30 (Table 2). The average Ka/Ks ratio measured within mammal, reptile and bird lineages was 0.13, 1.38 and 1.43, respectively.

**Table 2:** Natural selection and functional divergence parameters

Sequence	Method	Ka	Ks	Ka/Ks	Length	S-sites	N-sites	Substitutions	S-substitutions	N-Substitutions	Divergence rate	GC content (%)
Teleost PGI1/PGI2	NG	1.12	1.79	0.64	1624	370	1254	682	241	441	1.27	53
Teleost PGI1/PGI1	NG	0.10	1.37	0.07	1659	377	1282	354	236	118	0.39	54
Teleost PGI2/PGI2	NG	0.07	1.15	0.06	1659	375	1284	292	210	82	0.31	50
Teleost PGI1/Mammal PGI	NG	3.02	1.59	1.96	1682	392	1290	1202	256	945	2.68	56
Teleost PGI2/Mammal PGI	NG	2.60	2.11	1.30	1670	388	1282	1200	271	929	2.49	54
Reptile/Reptile PGI	NG	3.08	2.22	1.38	1851	432	1419	1354	307	1047	2.88	53
Mammal/MammaL PGI	NG	0.05	0.40	0.13	1674	391	1283	182	115	67	0.13	56
Bird/Bird PGI	NG	3.50	2.66	1.43	1569	360	1209	1155	258	897	3.31	47

The average divergence rate between teleost PGI1 and PGI2 paralogs was 1.27, which was higher than the rate between teleost PGI1 orthologs (0.38) and teleost PGI1 orthologs (0.31) (Table 2). The average divergence rate of teleost PGI1/mammal PGI and teleost PGI1/mammal PGI2 were 2.68, and 2.49, respectively. The average divergence rates within reptile (2.88) and bird (3.31) lineages were significantly higher than that within mammal lineage (0.13) (Table 2). The average GC content between teleost PGI1/PGI2 paralogs was 53 % whereas it was 54 % for teleost PGI1 orthologs and 50 % for PGI2 orthologs (Table 2). The comparison between teleost and mammal showed similar values 56 % for teleost PGI1/mammal PGI and 54 % for teleost PGI2/mammal PGI. The average GC content within lineages was 47 % for bird, 53 % for reptile and 56 % for mammal (Table 2). For all pairwise comparisons, no codon bias was found. The substitution-rate-ratio (SRR) was 1:1:1:1 for all pairwise PGI orthologs and paralogs.

### Secondary structure

The comparison of the secondary structure between species revealed a number of changes (including differences in the number of helices, strands and coils) that were observed between PGI1 and PGI2 after duplication (Additional file 2: Figure S1A). The number of α helices of PGI1 and PGI2 was respectively, 22 and 24, the number of β-strands 12 and 11 and the number of coils 36 and 35. The TMD has the same length and is comprised of 22 amino acid residues. A number of substitutions have also occurred in the TMD, including those at positions 1: T-S, 4: M-T, 7:A-V, 9:V-I and 18:V-I. Any of these substitutions were associated with changes in the electric charge of the corresponding amino acid. Thirteen substitutions resulted in electric charge changes in PGI1 and 7 amino acid replacements associated with changes of electric charges in PGI2 (Additional file 2: Figure S1B). Four residues were with an *RSA* value, 3 of which were common, and only one different and located at the position 18 of the TMD. There were more amino acid residues with *RSA* value coil and structure compared to helices (Additional file 2: Figure S1C).

## Discussion

The current study aimed at inferring the evolutionary history and functional divergence of PGI genes in vertebrates. Two paralogues were identified in teleost fishes and only one isoform in all other lineages. The phylogenetic reconstructions, which incorporated protein sequences from completely sequenced genome with sequences from individual genomic characterisation recovered five main lineage-specific groups supported by high bootstrap values. This phylogenetic topology is not only supported by the conserved synteny results, but also by the molecular evolution analyses based on the estimation of the average ratio of nonsynonymous to synonymous changes and the divergence rates. The combined phylogenetic reconstructions and synteny-based analyses revealed, as expected, that vertebrate PGI genes originated from two rounds of genome duplications and their functional diversification derived from both amino acid changes and post-duplication rearrangements including inversion, intron gain (e.g. insertion of a genomic fragment) or loss (e.g. by fusion of exons) followed by the fusion of adjacent exons.

### Origin and diversification of vertebrate PGI genes

The phylogenetic reconstructions, similarity and conserved synteny analyses allowed the identification of two PGI isoforms (PGI1 and PGI2) in teleost fishes, in agreement with previous findings [46, 47], and only one PGI gene in the other vertebrates including ray-finned fishes, amphibians, birds and mammals. The diversification of PGI gene into PGI1 and PGI2 has likely resulted from the additional WGD that has specifically occurred within the teleost lineage [52]. The phylogenetic tree partitioned vertebrate PGI genes into five main groups (lungfishes/coelacanth/cartilaginous fishes, teleosts, amphibians, reptiles/birds and mammals), which are also supported by the molecular evolution results (Ka/Ks ratios and divergence rates), and synteny-based analyses. Overall, groups identified with the phylogenetic reconstructions are also supported by the repartition of PGI genes on the PCA plot according to their intron length. These results are consistent with previous findings indicating that reptiles and birds together have similar intron length, which are different from intron size of mammal and teleost genes [53, 54]. The positioning of *D. rerio* (DR) and *P. marinus* (PM) PGI on the PCA plot out of the teleost group is also consistent with findings from previous studies that have demonstrated that the intron size has expanded in these species compared to other teleosts [55]. The introns are less constrained by natural selection than protein coding fragments of the genome and therefore evolve faster. This may explain why they allowed to differentiate PGI genes into lineage-specific groups. This is in agreement with the absence of well differentiated groups on the PCA map showing the distribution of PGI genes according to exon length.

Although this cannot be inferred from the phylogenetic results, the synteny analyses support that teleost PGI2 is common to the other vertebrates including lungfishes, lamprey and the coelacanth. This suggests that this gene might existed in the genome of common vertebrate ancestor. This contradicts previous findings that the first duplication that gave rise to the vertebrate common PGI gene has occurred at the origin of hagfishes (agnatha) [56]. The presence of a PGI gene similar to the vertebrate isoform in hagfishes (*E. yangi*) and cartilaginous fishes, the elephant shark (*C. milii*), which is closely related to the invertebrates PGI (*D. melanogaster*) strongly supports for the existence of a PGI locus in the common ancestor of all vertebrates.

The teleost PGI1 and PGI2 are not adjacent in the genome, and then did not result from tandem duplications. Instead, they may have resulted from the duplication of large genomic fragments. Although it was not possible to determine whether all teleost PGI paralogs are located on corresponding chromosomes because most of them are located on unordered scaffolds or unknown random chromosomes, medaka PGI1 and PGI2 were identified on two distinct chromosomes (6 and 3) with a high degree of synteny [57]. These two chromosomes are probably corresponding chromosomes that resulted from WGD [57]. These results suggest that the two teleost PGI paralogs may have resulted from teleost-specific WGD that took place after the divergence of teleosts and lungfishes from their common ancestor [3, 52, 58, 59]. This interpretation is in agreement with previous findings based on the observation that the phylogenic position of PGI duplication coincides with the estimated teleost-specific WGD [46, 47].

### Natural selection and functional divergence

The significantly higher average pairwise Ka/Ks ratio (0.64) between teleost PGI1 and PGI2 paralogs compared to teleost PGI1 (0.07) or PGI2 (0.06) orthologs implies that each of these isoforms is highly constrained by the function(s)that it plays within this lineage through an intense purifying selection. Purifying selection is supported by the average divergence rate between PGI1 and PGI2 paralogs, which was significantly higher than the average rate recorded for PGI1 or PGI2 orthologs (1.27 versus 0.38 and 0.31, respectively). By contrast, for each species within the teleost lineage, PGI1 and PGI2 paralogs seems subject to high synonymous substitution rates resulting from directional selection, in accordance with the high divergence rates of the PGI1 and PGI2 paralogs. The average pairwise ratio Ka/Ks measured in certain teleost species such as *P. formosa, G. morhua* and *D. labrax* were exceptionally high (1.50, 2.07 and 1.74, respectively). This strongly supports the hypothesis of positive selection due to higher synonymous changes that may have resulted from changes in amino acid composition, codon bias or increased mutation rate. The substitution rate ratios measured in this study indicated that there was no codon bias, and the percentage of GC content for all pairwise comparisons was around 54 %, suggesting that the higher Ka/Ks ratios recorded for these three species resulted from an increased mutation rate compared to the remaining species. Given the heterogeneity of environments inhabited by teleost fishes it can be expected that positive selection acts differently on duplicates between species [60]. Certain species colonise environments where others cannot inhabit because of environmental constraints. Therefore, genes essential to adaptation and survival to these environments might be differently constrained, which may explain the differences in Ka/Ks ratios amongst teleosts.

The low average Ka/Ks ratio (0.13) and divergence rate (0.13) within mammals could be indicative of a more recent divergence of PGI orthologs compared to the avian and reptilian lineages. They may also indicate that mammalian PGI orthologs have the same functions in this lineage, i.e. they are being constrained by their different functions. By contrast, the higher Ka/Ks ratios (1.38 and 1.43, respectively) in the reptile and bird lineages may indicate that PGI genes have evolved different or novel functions. This interpretation is strongly supported by the average divergence rate, which was respectively 22 and 25 times the divergence rate measured within the mammal lineage. It has been suggested that the multi-functionality of PGI genes in mammals resulted from gradual changes in amino acid sequences [47]. The conservation of amino acid structure and the electric charge of PGI proteins measured in this study revealed amino acid substitutions between PGI1 and PGI2, some of which were associated with changes in the electric charges of the corresponding residues. There was divergence in the electric charge of PGI amino acid residues after duplication, which occurred more frequently in PGI2 compared to PGI1. Such changes in protein structure can be interpreted as a neo- and/or subfunctionalization, driven by functional constraints differentially exerted on PGI1 and PGI2 isoforms. These new results on the secondary structure and amino acid properties of PGI1 and PGI2 corroborate previous findings [47]. Indeed, the divergent evolution of the electric charges of PGI duplicates have been shown to reflect the specialisation of PGI isoforms [46, 47].

### Genomic rearrangements after PGI duplication

The combined length of the five *D. melanogaster* PGI exons was equivalent to the total length of the 18 exons of fishes and other vertebrate PGI genes, suggesting that the lower number of PGI exons in this species may have resulted from intron deletion and the fusion of adjacent exons following introns loss. This interpretation is supported by the alignment of amino acid sequences of genes (Additional file 3: Figure S2), which showed that *D. melanogaster* PGI has a length similar to that of its analogue in the other lineages. The sequence similarity of *D. melanogaster* PGI with that of mammals, birds, reptiles and amphibians is also equivalent to similarities found between these lineages. These results strongly support that the lower number of introns in *D. melanogaster* PGI compared to other species resulted from intron loss followed by fusions of adjacent exons. On the other hand, as demonstrated by the alignment of gene sequences (Additional file 3: Figure S2), the lower number of exons (15 exons) of the GM PGI compared to the number found in GG, implies that some exons at the upstream part of the gene have been lost in this species. The lower number of exons (11 exons) of PM PGI may be seen as a result of sequence incompleteness materialised by a sequencing gap in the PGI nucleotide sequence of GG (Additional file 3: Figure S2). However, sequence alignments provide strong evidence that the lower number of exons of the PGI of this species results from the deletion of some exons at both upstream and downstream parts of the sequence. Interestingly, the E13 has a total length of 130 bp in all mammal species in except in *SS* (44 bp), which also has a E14 different exon length compared to other mammals (84 versus 77 bp). More importantly, the exons E15, E16, E17 and E18 of the *SS* PGI have respectively the same length as mammal E14, E15, E16 and E17. This provides strong evidence that the additional exon of this species resulted from an insertion of a genomic DNA fragment within E13, which has led to two different and shorter exons (E13 and E14). By contrast, the additional exon of *L. oculatus* seems to have resulted from a re-organisation of the whole gene after duplication. Indeed, the PGI exons in this species have a different length compared to other vertebrates. This is supported by the contradictory phylogenetic and synteny results that respectively identified the PGI of *L. oculatus* as PGI1 and PGI2. Such post duplication genomic rearrangements may also explain why the PGI of *L. oculatus* which a holostean is grouped together with teleost PGI1 and closely related to *D. labrax* in the phylogenetic tree.

The order of genes surrounding PGIs support the idea that complex genomic rearrangements have occurred after duplication of the genomic fragments harbouring the PGI genes. The micro-synteny around PGI is preserved in most of the species, but in some cases, the first or first two flanking genes were lost. The succession of the three first adjacent downstream flanking genes of the PGI gene in birds/reptiles/amphibians is *WTIP*-*UBA2*-*PDCD2L*, whereas in the mammals analysed, *WTIP* was not found, and the order of the two flanking genes was inverted (*PDCD2L*-*UBA2*). The most parsimonious explanation to this finding is that the genomic fragment harbouring *PDCD2L* and *UBA2* was inverted after duplication, probably after the split of mammals from other tetrapods. The *PDCD2L* gene was then lost in amphibians while both the *UBA2* and *PDCD2L* genes were lost in the teleost fishes. The re-annotation of the region surrounding PGI did not allow identifying any of these genes, indicating that their misidentification is not due to incomplete sequencing. These genes have been truly lost after duplication, as was the *HSD17B2* gene, which was lost in certain fish species after PGI1/PGI2 duplication, but was conserved in the other species. The downstream flanking gene of *O. latipes* PGI2 is the same as the upstream flanking gene of PGI1 of other teleosts such as *T. nigroviridis*, *G. morhua, D. rerio* and *A. mexicanus*, while its upstream flanking gene is the same as that PGI2 of the other species. The upstream flanking gene of *O. latipes* PGI1 is the same as that of the remaining teleost species, but its downstream flanking gene is *SI:DKEY*, which corresponds to the second downstream flanking gene of PGI1 in the other species. The first downstream gene was probably lost in this species, probably through post-duplication rearrangements. These are examples of micro-synteny conservation between paralogs while the sequence homology signals were lost in many cases.

## Conclusion

The combined similarity search, conserved synteny and phylogenetic reconstruction analyses conducted in this study allowed an exhaustive clarification of the evolutionary history of PGI genes in vertebrates. The phylogenetic reconstructions differentiated vertebrate PGI genes into different groups, which were also supported by the synteny-based results and the selective and divergence tests. The results further showed that one PGI isoform, teleost PGI2 is shared by all vertebrate species analysed. PGI2 might be involved in the same biochemical pathways or physiological networks in vertebrates. The conservation of amino acid structure and the electric charge of PGI proteins, together with the evolutionary analyses based on Ka/Ks ratios and divergence rates, supports a functional diversification of teleost PGI as previously suggested [47]. Glycolysis, which is the main pathway in which the PGI genes are involved, is an energy metabolic production resource common to all eukaryotic organisms. This may explain why one of the PGI duplicates, PGI2, is shared by all vertebrate species. The PGI isoform specific to teleost fishes may play specific functions within this lineage as evidenced by different selective pressures and divergence rates. These probable novel functions have to be identified and investigated.

## Methods

### Identification of PGI orthologs and their flanking genes

The protein and nucleotide sequences of PGI1 and PGI2 of *Mugil cephalus* published by Grauvogel et al. [45] were extracted from the GenBank database using the accession number provided by the authors. These well-characterised PGI sequences were blasted against the sea bass, *Dicentrarchus labrax* genome (http://seabass.mpipz.mpg.de/cgi-bin/hgGateway), which allowed the identification of two PGI isoforms in this species. Two PGI loci were considered as paralogs or orthologs when the two corresponding nucleotide or protein sequences match on aligned blocks with an average length of at least 80 % with ≥70 % identify. I thus performed synteny-based analyses which consisted of identifying the putative PGI exon–intron structure and the comparison of exon/intron length within and between the main vertebrate lineages from hagfishes to mammals. They also consisted on performing a comprehensive comparative analysis of the genomic region harbouring PGI genes. I also performed a re-annotation of the region potentially harbouring PGI genes when a PGI gene was not previously identified in a given species. Thus the genomic location on chromosomes or scaffolds, as well as their exact position in their genomic entities were determined. Their upstream and downstream flanking genes were then determined and when they were not previously identified, the genomic region potentially harbouring them was re-annotated. To identify the PGI orthologs in the other teleosts, the two isoforms identified in the sea bass genome were blasted against the genome of teleosts available in the Ensembl Genome Browser. The same criteria mentioned above (aligned blocks of protein sequences that match with an average length of at least 80 % with ≥70 % identity) were applied to identify real orthologs. The upstream and downstream genes of each PGI were identified in the other teleost species by similarity search using the nucleotide and protein sequences of genes that flank the PGIs in sea bass. The PGI orthologs of the other vertebrates were obtained from Ensembl by means of blast search using the teleost PGI sequences. When a PGI gene could not be identified in a given species and its the upstream and downstream flanking genes found, their sequences were used for PGI gene identification. Likewise, in cases where a PGI gene and none of its flanking genes were found in a species, the genomic DNA fragment harbouring PGI and the flanking genes was extracted and re-annotated using de novo and/or similarity-based annotation approaches. For the similarity-based annotations, a gene was considered as a PGI or flanking locus when it matched the well-characterised PGI sequences on aligned blocks with an average length of at least 80 % with ≥70 % identify. The protein and nucleotide sequences of predicted genes from the de novo annotation were confirmed as PGI or flanking loci by blast against the well characterised PGI genes using the above criteria. For the species whose genome has not been completely sequenced, the accession numbers of the PGI genes were obtained from the literature and then used to identify the corresponding sequence in GenBank. The information on exon–intron structure of each PGI locus whose genome is not available in the Ensembl Genome Browser was extracted from the transcript-summary table that can be downloaded from the Blast/Blat research output results.

### Sequence alignment, phylogenetic and principal component analyses

A phylogenetic tree was reconstructed using protein sequences of PGI genes of species belonging to the main vertebrate lineages. The protein sequences of all PGI isoforms identified in vertebrate species were aligned using MAFFT version 7 (http://mafft.cbrc.jp/alignment/server). The Gblocks Server (http://molevol.cmima.csic.es/castresana/Gblocks_server.html) was used to improve the alignment. The well-aligned blocks were then used to reconstruct a phylogenetic tree using MEGA software version 6. The maximum likelihood method with the Jones–Taylor–Thornton (JTT) substitution model was used to constructed the phylogenetic tree, which was rooted with the PGI protein sequences of the lancelet, *Branchiostoma floridae* and the hagfish, *Eptatretus yangi*. Principal component analysis (PCA) was performed on both PGI exon and intron length separately using the ade4 packages of the R software version v.64 3.1.1. Principal component analysis (PCA) was performed within a phylogenetic context on both PGI exon and intron lengths. Although not commonly used in the comparative genomics, it was considered particularly useful to illustrate the relationship between PGI genes based on their exon and intron lengths. Functional divergence between duplicates could be the results of changes in amino acid residues of the coding sequences, but it could also be related to changes in non-coding regions (including introns) which can lead to functional divergences between duplicates. It has been demonstrated that functional divergence can be caused by amino acid substitutions in coding sequences or alterations of exon/intron structure [61].

### Tests for selection

The nonsynonymous (dN) and synonymous (dS) ratio (dN/dS), also known as Ka/Ks ratio or ω was used to measure the evolutionary selective pressure exerted on genes. Pairwise comparisons of Ka/Ks ratios were thus conducted between teleost PGI1 and PGI2 paralogs and, between reptile/bird and mammal PGI. Pairwise comparisons were also conducted between PGI of the latter two groups and teleost PGI2. The intra-specific Ka/Ks ratios between PGI1-PGI2 paralog pairs was calculated in each teleost fish using Nei and Gojobori method implemented in Ka/Ks_Calculator software [62]. There are several methods incorporated in Ka/Ks software calculator for the estimation of Ka/Ks ratios, which include NG [63], LPB [64, 65], MLPB [66] MLWL [66] and YN [67]. All the above listed methods were tested and the results were not significantly different between them. Finally, the NG method was applied and a Fisher’s exact test was used to access the significance of Ka/Ks >1 and Ka/Ks < 1 as implemented in Ka/Ks_Calculator software. Multiple comparison Turkey test was used to evaluate the significance of differences in Ka/Ks ratios between PGI orthologs and paralogs. The same approach was also used to calculate the inter-specific Ka/Ks ratios for each pairwise of PGI2 orthologs from all vertebrate groups that are analysed including teleost fishes. The divergence times were calculated between all PGI paralogs and orthologs using nucleotide sequences.

### Secondary structure

The SABLE server (http://sable.cchmc.org/) was used for the functional annotation, which included finding the number of transmembrane domains, predicting the secondary structure, quantifying the relative solvent accessibility (*RSA*) of amino acid residues along the protein sequences, and identifying physico-chemical property profiles. The *RSA* represents the solvent-accessible surface areas normalised by the surface area of the residue in the unfolded state, and is used to measure the solvent surface accessible of amino acid residues in a protein. An *RSA* value of 0 means that the surface area is completely buried whereas an *RSA* value of 9 is indicative of a fully exposed surface area. The predicted structure were visualised using the POLYVIEW-2D viewer (http://polyview.cchmc.org).

## Availability of supporting data

All datasets supporting the results of this article are included in the article and its additional files.

## Additional files

10.1186/s13104-015-1683-x A: Exon count and their corresponding length of the PGI genes in different vertebrate species analysed in this study. B: Intron count of PGI genes and their corresponding length in the different species analysed in this study. The code given to the PGI genes in the table corresponds to the initials of the scientific name of each species, followed by a number, which refers to the isoform.
10.1186/s13104-015-1683-x A: Secondary structure of PGI1 and PGI2. B: Comparison of electric charges of amino acid residues between PGI1 and PGI2. C: Comparison of relative sovant accessibility (RSA) between PGI1 and PGI2).
10.1186/s13104-015-1683-x A: CLUSTAL multiple alignment amino acid sequences of Drosophila melanogaster PGI with mammalian (*MM: Macaca mulatta*), Avian (*GG: Gallus gallus*), reptile (*AC: Anolis carolinenesis*), amphibian (*DuM: Duttaphrynus melanostictus*) and teleost (*DR: Danio rerio*) PGIs. B: Multiple alignment of amino acid sequences *M. gallopavo* (*MG*) PGI with *G. gallus* (*GG*) PGI. C: Multiple alignment of nucleotide sequences P. marinus (*PM*) PGI with D. rerio PGI1 and PGI2.

## Abbreviations

PGI: phosphoglucose isomerase
*UFG2*: upstream flanking gen 1
UFG2: upstream flanking gene 2
*DFG1*: downstream flanking gene 1
*DFG2*: downstream flanking gene 2
*DFG3*: downstream flanking gene 3
*WGD*: whole genome duplications
MAFFT: Multiple Alignment using Fast Fourier Transform
JTT: Jones–Taylor–Thornton
PCA: principal component analysis
dN: nonsynonymous substitution
dS: synonymous substitution
SRR: Substitution-rate-ratio
*HSD17B2*: hydroxysteroid (17-beta) dehydrogenase 2
*LSM14AA*: LSM14A mRNA processing body assembly factor a
*MPHOSPH6*: M-phase phosphoprotein 6
*UBA2*: ubiquitin-like modifier activating enzyme 2
*PDCD2L*: programmed cell death 2-like gene
*WTIP*: wilms tumor 1 interacting protein
*DM*: *Drosophila melanogaster*
*LO*: *Lepisosteus oculatus*
*SS*: *Sus scrofa*
*AM*: *Astyanax mexicanus*
*GM*: *Gadus morhua,*
*PS*: *Pelodiscus sinensis*
*MP*: *Petromyzon marinus*
*PF*: *P. formosa*
*TR*: *Takifugu rubripes*
*TN*: *Tetraodon nigorviridis*
*LO*: *Lepisosteus oculatus*
*MG*: *Meleagris gallopavo*
*PS*: *Pelodiscus sinensis*
*LC*: *Latimeria chalumnae*
*AC*: *Anolis carolinensis*
*MM*: *Mus musculus*
*PA*: *Pongo abelii*
*PM*: *Petromyzon marinus*
