# Supplementary material for: Evolutionary significance and diversification of the phosphoglucose isomerase genes in vertebrates
Source: BMC Res Notes. 2015 Dec 18;8:799. doi: 10.1186/s13104-015-1683-x (PMC4684624; doi:10.1186/s13104-015-1683-x)
Supplement: Supplementary file 1 — 10.1186/s13104-015-1683-x A: Exon count and their corresponding length of the PGI genes in different vertebrate species analysed in this study. B: Intron count of PGI genes and their corresponding length in the different species analysed in this study. The code given to the PGI genes in the table corresponds to the initials of the scientific name of each species, followed by a number, which refers to the isoform. [file 13104_2015_1683_MOESM1_ESM.pdf]

Additional file 1: Table S1. A:

Code_PGI	E1	E2	E3	E4	E5	E6	E7	E8	E9	E10	E11	E12	E13	E14	E15	E16	E17
AP	140	91	69	120	84	147	72	45	54	61	44	153	130	77	129	76	67
AC	119	91	69	120	84	147	72	45	54	61	44	153	130	77	129	76	67
AM1	321	91	69	120	84	147	72	45	54	61	44	153	130	77	205	67	541
AM2	274	91	69	120	84	147	72	45	54	61	44	153	130	77	129	76	67
DR1	260	91	69	120	84	147	72	45	54	61	44	153	130	77	129	76	67
DR2	166	91	69	120	84	147	72	45	54	61	44	153	130	77	129	76	67
DM	637	583	240	529	471	0	0	0	0	0	0	0	0	0	0	0	0
EC	122	91	69	120	84	147	72	45	54	61	44	153	130	77	129	76	67
FC	119	91	69	120	84	147	72	45	54	61	44	153	130	77	129	76	67
FA	64	91	69	120	84	147	72	45	54	61	44	153	130	77	129	76	67
GM1	119	91	69	120	84	147	72	45	54	61	44	153	130	77	129	76	67
GM2	125	97	69	120	84	147	72	45	54	61	44	153	130	77	129	76	65
GG	171	91	69	120	84	147	72	45	54	61	44	153	130	77	129	76	67
GA1	167	91	69	120	84	147	72	45	54	61	44	153	130	77	129	76	67
GA2	157	91	69	120	84	147	72	45	54	61	44	153	130	77	129	76	67
HS	280	129	91	69	120	147	72	45	54	61	44	153	130	77	129	76	67
LC	718	91	69	120	84	147	72	45	54	61	44	153	130	77	129	76	67
LO	190	91	36	28	125	84	147	87	45	54	61	44	153	130	77	129	76
MM	211	91	69	120	84	147	72	45	54	61	44	153	130	77	129	76	67
MG	130	84	147	72	45	54	61	44	153	130	77	129	76	67	437	0	0
MMc	190	91	69	120	84	147	72	45	54	61	44	153	130	77	129	76	67
ON1	128	91	69	120	84	147	72	45	54	61	44	153	130	77	129	76	67
ON2	242	91	69	120	84	147	72	45	54	61	44	153	130	77	129	76	67
OL1	205	91	69	120	84	147	72	45	54	61	44	153	130	77	129	76	67
OL2	119	91	69	120	84	147	72	45	54	61	44	153	130	77	129	76	67
PS	21	69	120	84	147	72	45	54	61	44	153	130	77	129	76	67	4135
PM	284	75	84	120	84	147	72	45	54	61	44	0	0	0	0	0	0
PF1	302	91	69	120	84	147	72	45	54	61	44	153	130	77	129	76	67
PF2	258	91	69	120	84	147	72	45	54	61	44	153	130	77	129	76	67
PA	973	91	69	120	84	147	72	45	54	61	44	153	130	77	129	76	67
RN	188	91	69	120	84	147	72	45	54	61	44	153	130	77	129	76	67
SS	122	91	69	120	84	147	72	45	54	61	44	153	44	84	79	129	76
TF1	170	91	69	120	84	147	72	45	54	61	44	153	130	77	129	76	67
TF2	148	91	69	120	84	147	72	45	54	61	44	153	130	77	129	76	67
TN1	108	69	120	84	147	72	45	54	61	44	153	105	22	54	54	71	0
TN2	145	91	64	128	84	153	72	45	54	61	44	153	130	77	129	90	0
XM1	192	91	69	120	84	147	72	45	54	61	44	153	130	77	129	76	67
XM2	357	91	69	120	84	147	72	45	54	61	44	153	130	77	129	76	67

Additional file 1: Table S1. B:

Code_PGI	In1-2	In2-3	In3-4	In4-5	In5-6	In6-7	In7-8	In8-9	In9-10	In10-11	In11-12	In12-13	In13-14	In14-15	In15-16	In16-17	In17-18
AP	3526	897	1870	946	83	1735	1050	793	964	1601	243	360	366	649	539	85	1529
AC	10037	521	3889	533	803	4559	2449	1082	6127	1837	943	1359	1833	103	1799	98	2317
AM1	2263	929	414	89	751	567	171	849	290	165	290	382	609	517	116	182	
AM2	1855	188	1179	511	1528	1358	178	1634	1296	115	296	1541	808	1742	1153	1393	520
DR1	3054	2385	1842	2269	608	438	944	83	125	1609	361	2437	963	693	1914	2133	1425
DR2	1286	82	1476	103	2337	82	83	1447	1817	75	2574	2100	1900	2198	1843	103	460
DM	245	348	62	55													
EC	1002	358	1433	4394	134	769	497	1285	10468	409	134	949	153	1449	220	85	111
FC	451	357	1429	4568	129	876	502	1313	10514	416	104	1184	156	1051	217	85	105
FA	8725	366	3013	1830	85	3180	998	773	789	1623	220	308	367	1262	411	79	1637
GM1	974	3025	652	1399	1368	602	165	139	264	136	308	418	134	211	298	327	411
GM2	357	2225	1275	325	637	528	217	1985	553	149	135	741	490	151	387	346	0
GG	3716	857	2548	1214	85	1693	987	791	971	1644	221	356	351	1337	558	83	1387
GA1	1419	1107	1857	75	86	314	317	194	109	144	100	171	78	79	127	233	78
GA2	246	239	228	84	183	129	85	96	525	191	125	162	140	211	231	254	204
HS	240	956	347	1731	9032	1052	512	1903	11728	419	142	2234	150	2549	220	87	115
LC	3567	2170	866	3415	18441	4463	1692	858	3214	861	1972	2175	918	1362	1650	94	2047
LO	1204	809	2780	163	487	687	64	1252	721	825	1338	163	404	521	481	1247	487
MM	953	346	2053	9821	148	1029	513	1909	12929	717	144	2214	148	3140	220	87	110
MG	1166	88	1696	1009	766	972	1640	222	348	354	1155	543	85	1924	0	0	0
MMc	718	286	1690	6191	82	1511	688	2288	7645	349	98	1670	161	1410	1138	111	87
ON1	851	264	870	74	77	122	139	291	424	224	271	160	761	107	110	126	487
ON2	208	221	208	166	93	112	89	82	755	241	163	113	705	1545	1336	491	447
OL1	657	212	72	74	104	216	752	2092	1768	78	225	319	272	124	69	160	68
OL2	158	214	74	86	74	1694	86	92	538	1031	104	88	196	320	1349	347	145
PS	1522	2867	2257	91	1973	1559	1266	1652	1228	909	1103	404	1464	913	80	1546	
PM	4808	3507	1899	1143	564	256	1044	364	921	1048							
PF1	1461	1412	92	144	192	80	70	197	267	1074	921	83	79	754	74	120	251
PF2	174	150	98	85	1355	148	85	76	1283	316	111	1107	330	238	236	806	1212
PA	959	347	1725	10298	147	1049	507	1917	11988	420	142	2227	149	2643	220	87	110
RN	755	252	2055	6160	82	1510	722	1705	7537	307	93	1782	161	1312	1149	197	84
SS	976	337	1627	7601	159	994	735	1314	10738	414	142	889	106	51	2696	219	76
TF1	1079	396	112	76	74	128	88	186	172	168	73	87	67	67	141	289	73
TF2	299	161	199	139	78	522	87	123	471	310	83	76	262	307	223	1877	220
TN1	316	68	66	64	90	74	75	170	108	103	75	4	60	2101	99	0	0
TN2	155	160	185	141	74	551	78	125	482	346	79	133	194	373	218	0	0
XM1	182	146	107	85	1354	157	87	76	1282	313	120	1215	319	227	238	821	279
XM2	1489	1503	85	108	201	80	70	139	260	1156	886	83	79	765	79	121	297
