# Supplementary figures and images for: Evolutionary significance and diversification of the phosphoglucose isomerase genes in vertebrates
Source: BMC Res Notes. 2015 Dec 18;8:799. doi: 10.1186/s13104-015-1683-x (PMC4684624; doi:10.1186/s13104-015-1683-x)

Additional file 2: Figures S1. A:

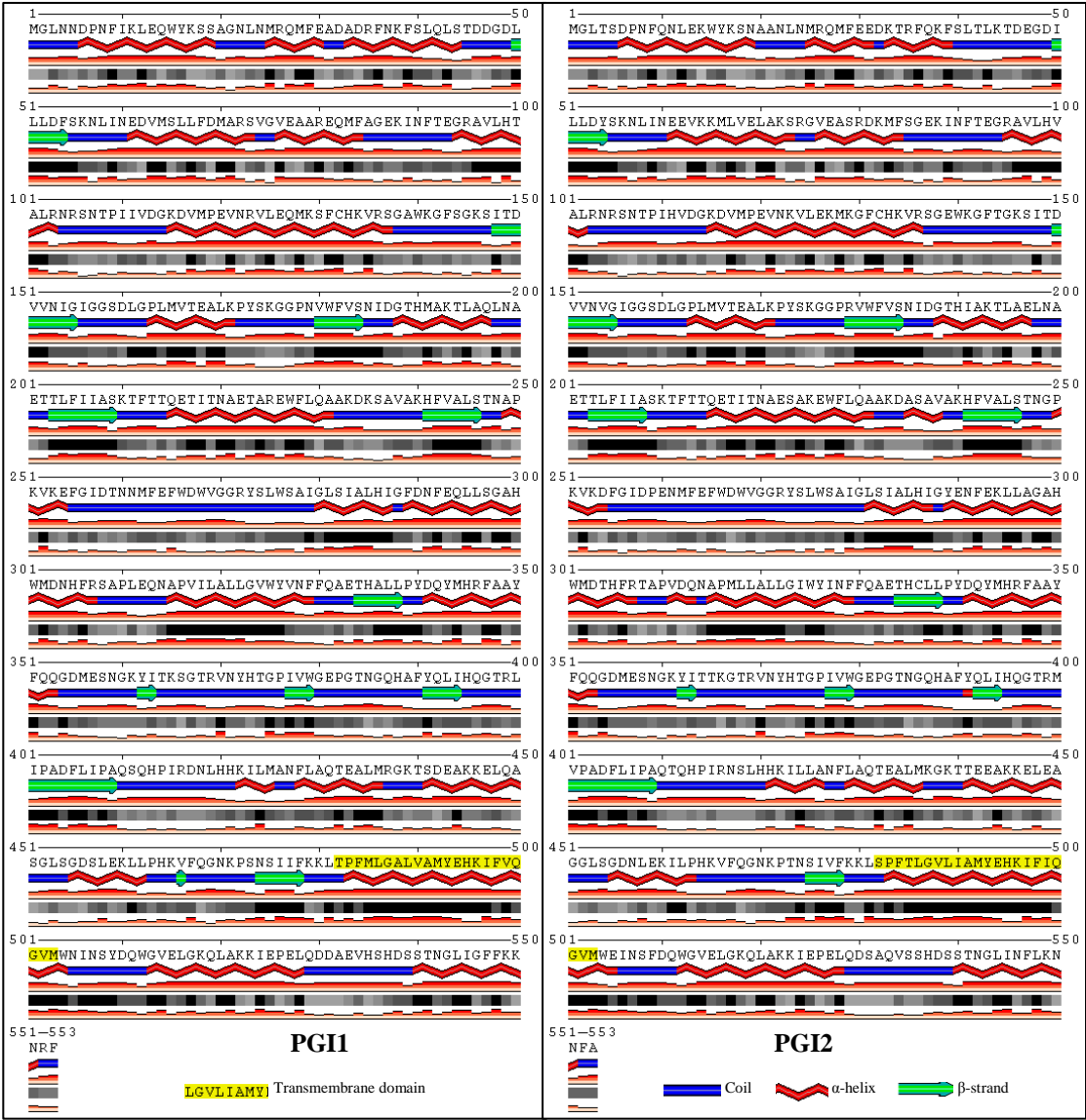

## Additional file 2: Figures S1. B:

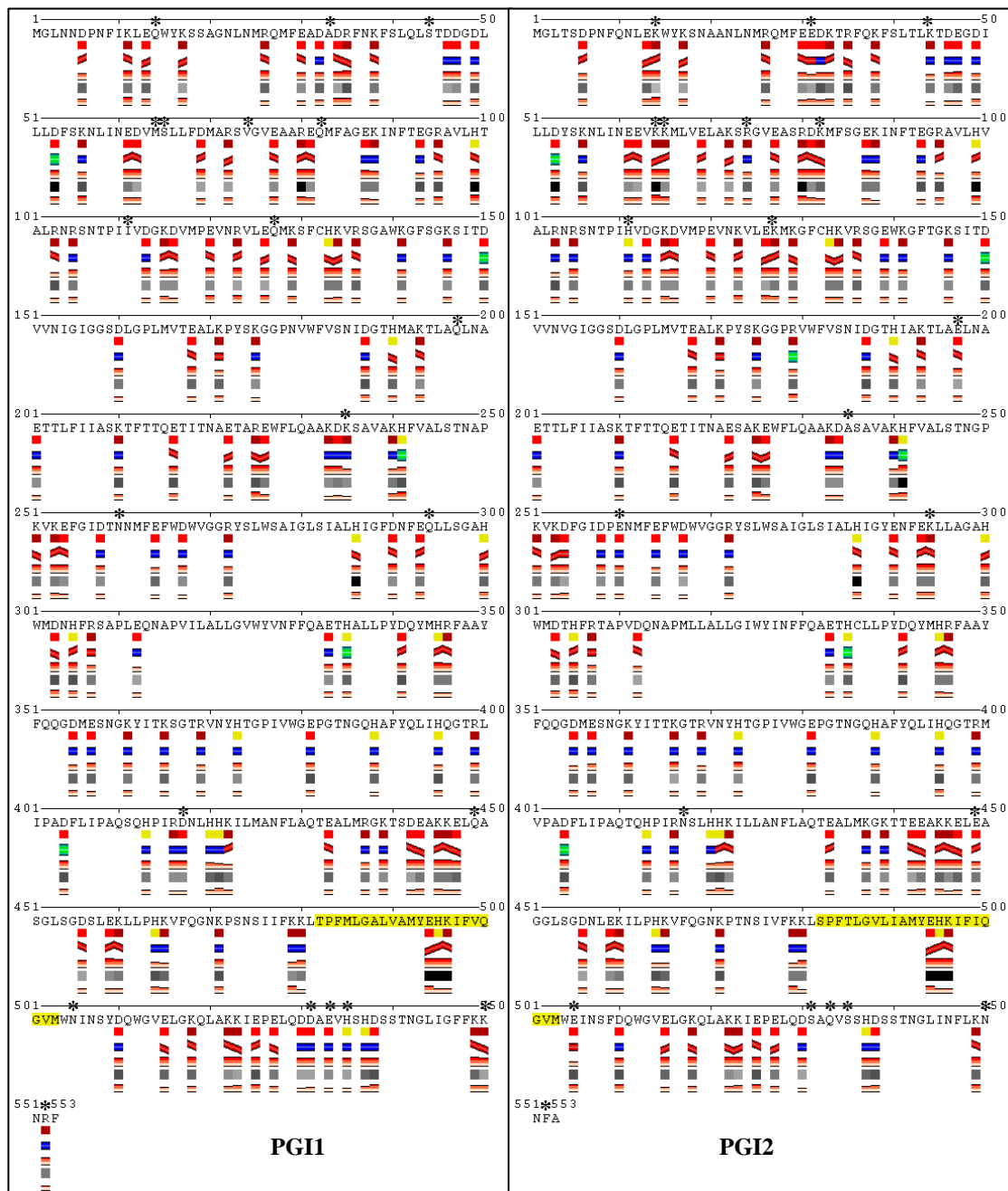

## Additional file 2: Figures S1. C:

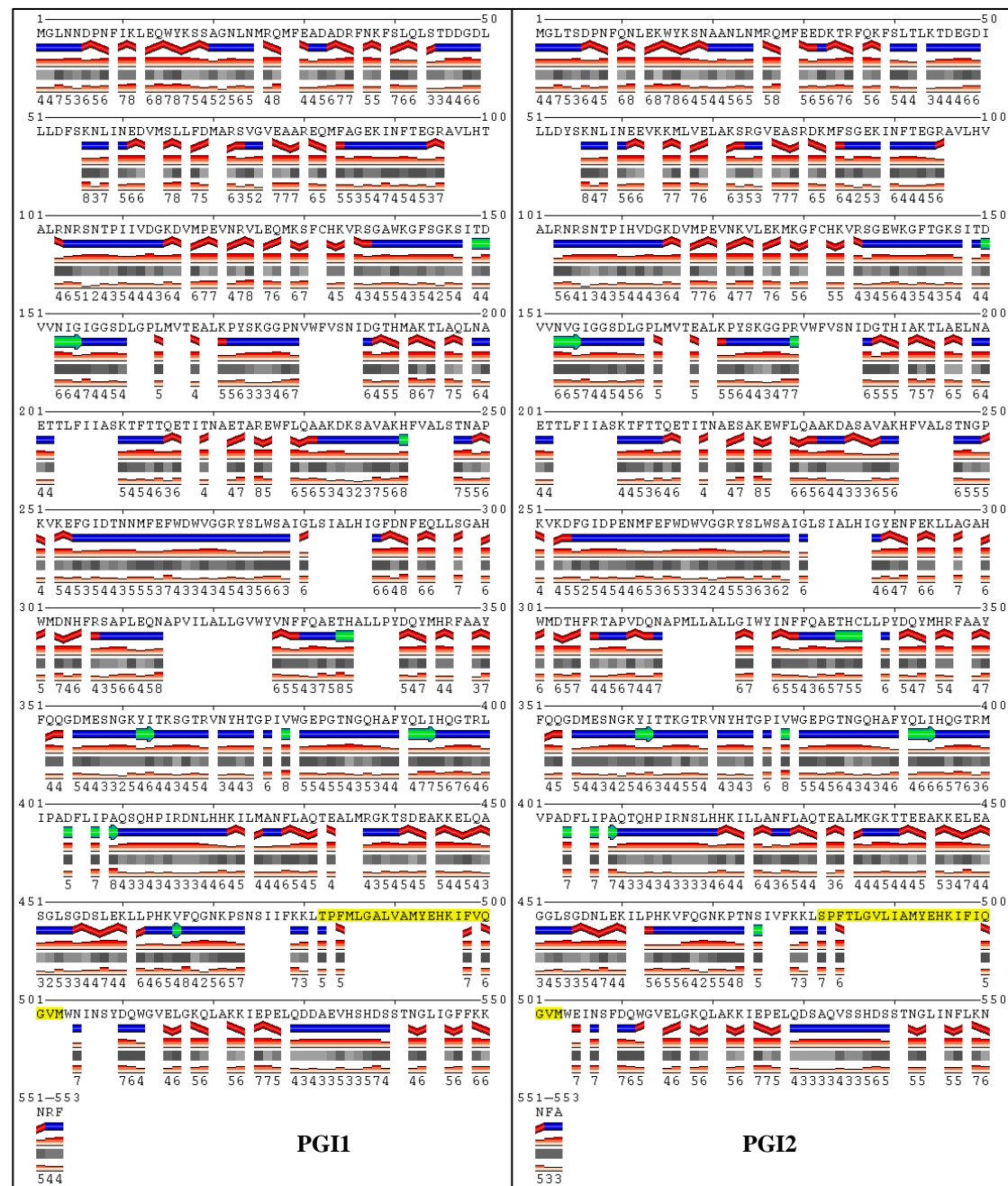

Supplement: Supplementary file 2 — 10.1186/s13104-015-1683-x A: Secondary structure of PGI1 and PGI2. B: Comparison of electric charges of amino acid residues between PGI1 and PGI2. C: Comparison of relative sovant accessibility (RSA) between PGI1 and PGI2). [file 13104_2015_1683_MOESM2_ESM.pdf]
