# Supplementary material for: Evolutionary significance and diversification of the phosphoglucose isomerase genes in vertebrates
Source: BMC Res Notes. 2015 Dec 18;8:799. doi: 10.1186/s13104-015-1683-x (PMC4684624; doi:10.1186/s13104-015-1683-x)
Supplement: Supplementary file 3 — 10.1186/s13104-015-1683-x A: CLUSTAL multiple alignment amino acid sequences of Drosophila melanogaster PGI with mammalian (MM: Macaca mulatta), Avian (GG: Gallus gallus), reptile (AC: Anolis carolinenesis), amphibian (DuM: Duttaphrynus melanostictus) and teleost (DR: Danio rerio) PGIs. B: Multiple alignment of amino acid sequences M. gallopavo (MG) PGI with G. gallus (GG) PGI. C: Multiple alignment of nucleotide sequences P. marinus (PM) PGI with D. rerio PGI1 and PGI2. [file 13104_2015_1683_MOESM3_ESM.pdf]

Additional file 3: Figures S2. A

```
DR_MGI1_      -----MGLNNDPNFIKLEQWYKSSAGNLNMRQMFEADADRFNKFSLQLST-DDGDLLLDF
GG_PGI__      -----MPLSADPHFKKLLEWHKANASKLVLRQLFEADKDRFHKFSLTLNT-DHGDILLDY
DM_PGI__      MAGPLPPLNQEAAFQKLQEYYDSKGKDLNIKDLFVKDSKRFSKYSLRLHTQNDGEILLDY
MM_PGI__      ----MAALTRDPQFQKLQQWYREHGSELNLRRLFDADKDRFNHFSLTLNT-NHGHILLDY
DR_PGI2_      -----MGLTSDPNFQNLEKWYKSNAANLNMRQMFEEDKTRFQKFSLTLKT-DEGDILLDY
AC_PGI__      -----MTLSSDAAFQKLREWHKAHAAQLVLRQLFDADKDRFRKFSLTLNT-DQGDILVDY
DuM_PGI_      -----MALTSDPVYQKLAKFYEANHGCLNLRKLFEADKERFNKFSKTLNT-GEGDILVDY

DR_MGI1_      SKNLINEDVMSLLFDMARSVGVEAAREQMFAGEKINFTEGRAVLHTALRNRSNTPIIVDG
GG_PGI__      SKNLVTEEVMKMLIELAKSRGVESARERMFSGEKINFTENRAVLHIALRNRSNTPILVDG
DM_PGI__      SKNRINDEVWDLLLTLAKVRRVNAARDAMFSGQHINITENRAVLHTALRNRGTDPVLVDD
MM_PGI__      SKNLVTEDVMRMLVDLAKSRGVEAARERMFNGEKINYTEGRAVLHVALRNRSNTPILVDG
DR_PGI2_      SKNLINEEVKKMLVELAKSRGVEASRDKMFSGEKINFTEGRAVLHVALRNRSNTPIHVDG
AC_PGI__      SKNLVTEDVMKLLIDVAKSRGIEKAREQMFSGEKINFTENRAVLHIALRNRSNTPILVDG
DuM_PGI_      SKNLITEEVIKLLIELARSRGVEAARKRMFSAEKINFTENRAVLHIALRNRSNAPIQVDG

DR_MGI1_      KDVMPEVNRVLEQMKSFCHKVRSGAWKGFSGKSITDVVNIGIGGSDLGPLMVTEALKPYS
GG_PGI__      KDVVPEVNKVLDKMKHFCQKVRSGEWKGYTGKAITDVVNIGIGGSDLGPLMVTEALKPYS
DM_PGI__      KDVMPDVRAELAHMKEFTNMVISGVWRGCTGKQITDVVNIGIGGSDLGPLMVTEALKPYG
MM_PGI__      KDVMPEVNKVLDKMKSFCQRVRSGDWKGYTGKTITDVINIGIGGSDLGPLMVTEALKPYS
DR_PGI2_      KDVMPEVNKVLEKMKGFCHKVRSGEWKGFTGKSITDVVNVGIGGSDLGPLMVTEALKPYS
AC_PGI__      KDVVPDVNKVLEKMKGFCQKVRSGDWKGYSGKSMTDVVNVGIGGSDLGPLMVTEALKPYS
DuM_PGI_      KDMMPEVNAVLGKMKTFCQKVRSGDWKGYSGKAITDVVNVGIGGSDLGPLMVTEALKPYS

DR_MGI1_      KGGPNVWFVSNIDGTHMAKTLAQLNAETTLFIIASKTFTTQETITNAETAREWFLQAAKD
GG_PGI__      KGGPRVWFVSNIDGTHIAKTLAELHPETTLFIIASKTFTTQETITNAVTAKEWFLHAAKD
DM_PGI__      KG-LHSHFVSNIDGTHLAEVLKKVNYETTLFIVASKTFTTQETITNATSAKTWLLEHSKE
MM_PGI__      SEGPRVWYVSNIDGTHIAKTLTQLNPESSLFIIASKTFTTQETITNAETAKEWFLQAAKD
DR_PGI2_      KGGPRVWFVSNIDGTHIAKTLAELNAETTLFIIASKTFTTQETITNAESAKEWFLQAAKD
AC_PGI__      KGGPRVWFVSNIDGTHMAKTLAELNPETTLFIIASKTFTTQETITNAETAKEWFLKAAKD
DuM_PGI_      KGGPRVWFVSNIDGTHMAKTLAELNPETTLFIVASKTFTTQETITNAETAKEWFLKAAKD

DR_MGI1_      KSAVAKHFVALSTNAPKVKEFGIDTNNMFEFWDWVGGRYSLWSAIGLSIALHIGFDNFEQ
GG_PGI__      PSAVAKHFVALSTNGPKVKEFGIDTENMFEFWDWVGGRYSLWSAIGLSIALHIGFDNFES
DM_PGI__      PESVAKHFVALSTNKEKVTEFGIDSTNMFGFWDWVGGRYSLWSAIGLSICLSIGFENFEQ
MM_PGI__      PSAVAKHFVALSTNTTKVKEFGIDPQNMFEFWDWVGGRYSLWSAIGLSIALHVGFDNFEQ
DR_PGI2_      ASAVAKHFVALSTNGPKVKDFGIDPENMFEFWDWVGGRYSLWSAIGLSIALHIGYENFEK
AC_PGI__      PGAVAKHFVALSTNGPKVKDFGIDTQNMFEFWDWVGGRYSLWSAIGLSIALHIGYDNFEK
DuM_PGI_      PSAVAKHFVALSTNAPKVKDFGIDTANMFEFWDWVGGRYSLWSAIGLSIALHVGFDNFEK

DR_MGI1_      LLSGAHWMDNHFRSAPLEQNAPVILALLGVWYVNFFQAETHALLPYDQYMHRFAAYFQQG
GG_PGI__      LLAGGHWMDKHFHTAPLEKNVPVLLAMLGVWYINCYGCETHALLPYDQYMHRFAAYFQQG
DM_PGI__      LLDGAHFMDNHFKTTPFEKNAPVILALLGVWYSNFFKAETHALLPYDQYLHRFAAYFQQG
MM_PGI__      LLSGAHWMDQHFRTTPLEKNAPVLLALLGIWYINCFGCETHAMLPYDQYLHRFAAYFQQG
DR_PGI2_      LLAGAHWMDTHFRTAPVDQNAPMLLALLGIWYINFFQAETHCLLPYDQYMHRFAAYFQQG
AC_PGI__      LLAGAHWMDNHFRTTPLEKNVPVVLAMLGVWYINCYGTETHALLPYDQYMHRFAAYFQQG
DuM_PGI_      LLAGAHWMDNHFCNTPLENNIPVILAMLGVWYINFYGCETQALLPYDQYMHRFAAYFQQG

DR_MGI1_      DMESNGKYITKSGTRVNYHTGPIVWGEPGTNGQHAFYQLIHQGTRLIPADFLIPAQSQHP
GG_PGI__      DMESNGKYITKKGSRVDYNTGPIVWGEPGTNGQHAFYQLIHQGTRMIPCDFMIPVQTQHP
DM_PGI__      DMESNGKFVSKSGKPVKYSTGPIVWGEPGTNGQHAFYQLIHQGTRLIPCDFIAPAQTHNP
MM_PGI__      DMESNGKYITKSGTRVDHQTGPIVWGEPGTNGQHAFYQLIHQGTKMIPCDFLIPVQTQHP
DR_PGI2_      DMESNGKYITTKGTRVNYHTGPIVWGEPGTNGQHAFYQLIHQGTRMVPADFLIPAQTQHP
AC_PGI__      DMESNGKYITKKGTRVNYSTGPIVWGEPGTNGQHAFYQLIHQGTRMIPCDFLIPVQTQNP
DuM_PGI_      DMESNGKYITKTGARVNYNTGPVVWGEPGTNGQHAFYQLIHQGTRMIPCDFMIPAQSQNP

DR_PGI1_      IRDNLHHKILMANFLAQTEALMRGKTSDEAKKELQASGLSGDSLEKLLPHKVFQGNKPSN
GG_PGI__      VRNGLHHKILLANFLAQTEALMKGKTADEARKELQAAGLSGDALEKLLPHKVFEGNRPTN
DM_PGI__      IAGGKHHKILLSNFLAQTEALMAGKTVDEARTELSKAGLCGNELDNLLPHKVFVGNRPTN
```

MM_PGI__ IRKGLHHKILLANFLAQTEALMRGKSTDEARKELQAAGKSPEDLERLLPHKVFEGNRPTN
DR_PGI2_ IRNSLHHKILLANFLAQTEALMKGKTTEEAKKELEAGGLSGDNLEKILPHKVFQGNKPTN
AC_PGI__ IRNGLHHKILLANFLAQTEALMKGKTTEEARQELQAAGMSGDALEKLLPHKVFEGNRPTN
DuM_PGI_ IRDGLHHKILMANFLAQTEALMKGKSTEEAKAELQASGLSGEALEKLLPHKVFEGNRPTN

Transmembrane domain

DR_PGI1_ SIIFKKL**TPFMLGALVAMYEHKIFVQGVM**WNINSYDQWGVELGKQLAKKIEPELQDDAEV
GG_PGI__ SIMFTKL**NPFTLGAIIAMYEHKIFVQGVV**WDINSYDQWGVELGKQLAKKIEPELESDAPV
DM_PGI__ SIVVKKV**SPFTLGALIALYEHKIFVQGII**WDINSFDQWGVELGKQLAKAIEPELDHCNEV
MM_PGI__ SIVFTKL**TPFMLGALVAMYEHKIFVQGII**WDINSFDQWGVELGKQLAKKIEPELDGSAQV
DR_PGI2_ SIVFKKL**SPFTLGVLIAMYEHKIFIQGVM**WEINSFDQWGVELGKQLAKKIEPELQDSAQV
AC_PGI__ SIMFTKL**NPFILGALIAMYEHKIFVQGVV**WDINSYDQWGVELGKQLAKKIEPELSSDAPV
DuM_PGI_ SIVFGKL**SPFILGALIAMYEHKIFVQGVV**WDINSYDQWGVELGKQLAKKIEPELESNAPV

DR_PGI1_ HSHDSSTNGLIGFFKKNRF----
GG_PGI__ TSHDSSTNGLISFIKKHRA----
DM_PGI__ STHDSSTNGLINFIKANWK----
MM_PGI__ TSHDASTNGLINFIKQQREARVQ
DR_PGI2_ SSHDSSTNGLINFLKNNFA----
AC_PGI__ TSHDSSTNGLIGFIQKHRA----
DuM_PGI_ TSHDSSTNGLINFYKAHRL----

Additional file 3: Figures S2. B

```
M.gallopavo   ------------------------------------------------------------
G. gallus     MPLSADPHFKKLLEWHKANASKLVLRQLFEADKDRFHKFSLTLNTDHGDILLDYSKNLVT

M.gallopavo   --------------------------------QNRAVLHIALRNRSNTPILVDGKDVVPE
G. gallus     EEVMKMLIELAKSRGVESARERMFSGEKINFTENRAVLHIALRNRSNTPILVDGKDVVPE

M.gallopavo   VNKVLDKMKHFCQKVRSGEWKGYTGKAITDVVNIGIGGSDLGPLMVTEALKPYSKGGPRV
G. gallus     VNKVLDKMKHFCQKVRSGEWKGYTGKAITDVVNIGIGGSDLGPLMVTEALKPYSKGGPRV

M.gallopavo   WFVSNIDGTHIAKTLAELHPETTLFIIASKTFTTQETITNAVTAKEWFLHAAKDPSAVAK
G. gallus     WFVSNIDGTHIAKTLAELHPETTLFIIASKTFTTQETITNAVTAKEWFLHAAKDPSAVAK

M.gallopavo   HFVALSTNGPKVKEFGIDTENMFEFWDWVGGRYSLWSAIGLSIALHIGFDNFESLLAGGH
G. gallus     HFVALSTNGPKVKEFGIDTENMFEFWDWVGGRYSLWSAIGLSIALHIGFDNFESLLAGGH

M.gallopavo   WMDKHFHTAPLEKNVPVLLAMLGVWYINCYGCETHALLPYDQYMHRFAAYFQQGDMESNG
G. gallus     WMDKHFHTAPLEKNVPVLLAMLGVWYINCYGCETHALLPYDQYMHRFAAYFQQGDMESNG

M.gallopavo   KYITKKGSRVDYNTGPIVWGEPGTNGQHAFYQLIHQGTRMIPCDFMIPVQTQHPIRNGLH
G. gallus     KYITKKGSRVDYNTGPIVWGEPGTNGQHAFYQLIHQGTRMIPCDFMIPVQTQHPVRNGLH

M.gallopavo   HKILLANFLAQTEALMKGKTADEARKELQAAGLSGDALEKLLPHKVFEGNRPTNSIMFTK
G. gallus     HKILLANFLAQTEALMKGKTADEARKELQAAGLSGDALEKLLPHKVFEGNRPTNSIMFTK

M.gallopavo   LNPFTLGAIIAMYEHKIFVQGVVWDINSYDQWGVELGKQLAKKIEPELESDAAVTSHDSS
G. gallus     LNPFTLGAIIAMYEHKIFVQGVVWDINSYDQWGVELGKQLAKKIEPELESDAPVTSHDSS

M.gallopavo   TNGLISFIKKHRA
G. gallus     TNGLISFIKKHRA
```

Additional file 3: Figures S2. C

```
D. rerio PGI1   ------------------------------------------------------------
D. rerio PGI2   ------------------------------------------------------------
P. marinus      tttcgcttctttctctctcccgctgggttagcgcttgttgctttcgcacacgaaacgccg


D. rerio PGI1   ------------------------------------------------------------
D. rerio PGI2   ------------------------------------------------------------
P. marinus      cagccgccgcctctcgctttctctctctctccgtctttccgtgccttcatctcttcttag


D. rerio PGI1   ---------------------------------------------atgggcctcaacaac
D. rerio PGI2   ---------------------------------------------atgggactgacaagc
P. marinus      ccaccagcgagtgaaggaggagggctcgtcgcaatggcaagcgcaaccgggctgacgagc
                                                             *. ** ** *  *.*

D. rerio PGI1   gacccgaacttcatcaaactggagcaatggtacaaatccagcgcgggaaacctcaacatg
D. rerio PGI2   gacccgaacttccagaatctggagaaatggtacaaatccaacgccgccaatctcaacatg
P. marinus      gaccctgcgtggaagcgactgggcgagtggcacgccaagcattcggcctcgctcaagatg
                ***** .  *      . ****.  *.***.**.      .. * *     ***** ***

D. rerio PGI1   aggcaaatgttcgaggccgatgctgacagattcaacaagttcagtttgcagctgtcgaca
D. rerio PGI2   aggcagatgttcgaggaggataaaacccgattccaaaagttcagcctaacattaaaaaca
P. marinus      agacagctcttcgacaaggacgccgaacgattcgccaaattcagtctgctgctcaacact
                **.**. * ***** .  **..  .   *****   **.*****..*.  ..*    **

D. rerio_PGI1   gatgatggggatctcctgctggacttctccaaaaacctgatcaatgaagacgtcatgagt
D. rerio_PGI2   gacgaaggagatatttgctggattactccaaaaacctcatcaatgaagaagttaagaag
P. marinus      gatgacggagagctactgctggactactccaagaacctcatcacaccggaggnnnnnnnc
                **.** **.**  * .*******.* ******.***** ****    .** *--------
                                                                      gap
D. rerio PGI1   ttgctcttcgacatggcgagatcagtgggcgtcgaggcggcccgggaacagatgtttgca
D. rerio PGI2   atgctcgtggagctggcaaagtctcgaggcgtggaggcgtccagagacaagatgttttca
P. marinus      gtccttgc-gttcaggcacgttcacgtggaattgaaaaggccagagaccgcatgttcagc
                 * **. . *    ***. . **    ** .* **.. * ** *.**  . *****.

D. rerio PGI1   ggagagaagatcaacttcactgagggtcgtgccgtcctccacaccgccctgaggaaccgc
D. rerio PGI2   ggagaaaagattaatttcactgagggtcgtgctgttctccatgtggctctgaggaaccgc
P. marinus      ggggagaagattaacttcacagagaaccgcgcggtgctgcacgtggctttgcgcaaccgc
                **.**.*****.**.***** ***...**.** ** ** **... **..** * ******

D. rerio PGI1   tccaacactcccatcatagtggacggaaaagacgtgatgccagaggtgaaccgagtcctg
D. rerio PGI2   tcaaacactcctatacatgtggacgggaaagacgtgatgccggaggtcaacaaggttctg
P. marinus      tccagcaagccgatggtggtggacggcaaggacgtgatgcccgacgtcaacgccgtgctg
                ** *.**  ** **    ******** **.*********** ** ** ***   ** ***

D. rerio PGI1   gagcagatgaagagcttctgccataaagtgcgcagcggcgcttggaaaggcttcagtggg
D. rerio PGI2   gagaagatgaaaggattctgtcataaagtccgcagtggcgagtggaagggattcactggg
P. marinus      gaaaagatgcgcggcttctgcgagagggtgcggagcggacagtggaagggctactctggc
                **. ***** . .* *****. * *..** ** **.**    *****.** * *  ***

D. rerio PGI1   aaaagcatcactgatgtggtgaacatcggcatcggaggatcagacctgggtcctctgatg
D. rerio PGI2   aaatccatcacggatgtggttaatgtgggcattggtggatctgatctgggtccgctaatg
P. marinus      aagccaatcgccgacgtcgtcaacatcggcatcggcggctctgacctgggccccctgatg
                **.   ***.* **.** ** **..* *****.** ** ** **.*****.** **.***

D. rerio PGI1   gtgactgaagcgctgaagccgtactctaaaggaggacccaatgtctggttcgtctctaat
D. rerio PGI2   gtgaccgaggctctgaagccgtactctaaaggcggcccgcgggtctggtttgtgtcaaac
P. marinus      gtgaccgaggcgctgaagccgtactcgaagggcggccccaactcgtggttcgtgtcgaac
                *****.**.** ************** **.** ** **  .  . *****.** ** **.
```

```
D. rerio PGI1   attgacggcacacacatggccaaaaccctcgcgcagctcaacgccgagaccacactcttc
D. rerio PGI2   attgacgggacacacatcgctaaaactctggctgaactcaacgctgagaccacactcttc
P. marinus      atcgacggcacgcacatggccaagacgctcgccgtgctcgaccccgagacgacgctcttc
                **.***** **.***** **.**.** ** **   .***.** *.***** **.******

D. rerio PGI1   atcatcgcctccaagacattcaccacccaggagaccatcactaacgcagagacggccaga
D. rerio PGI2   atcatcgcatccaagacattcaccactcaggaaaccatcacaaacgctgaatctgccaaa
P. marinus      atcattgcgtccaagacgttcacgactcaggagacgatcaccaacgccgagtcggccaag
                *****.** ********.***** **.*****.** ***** ***** **. * ****..

D. rerio PGI1   gaatggtttctgcaggccgctaaagacaaatctgctgtggccaaacatttcgtggctctt
D. rerio PGI2   gagtggtttctgcaggccgctaaagacgcatcagcagtagccaagcattttgtggcgctc
P. marinus      gagtggttcctgaagaaagctggagaccccgcggcagtcgcgaagcacttcgtggcactc
                **.*****.*** **.  ***..****    * ** ** ** **.**.**.***** **.

D. rerio PGI1   tccacaaacgcgccaaaagtgaaggagttcggcatcgacaccaacaacatgtttgagttt
D. rerio PGI2   tctacaaatggacccaaagtgaaggacttcggcatcgacccggagaacatgtttgagttc
P. marinus      tccacaaacggggaaaaagtgaaggctttcggcatcgacacaaacaacatgttcgagttc
                **.*****.* .   **********  ************ * .* ********.*****.

D. rerio PGI1   tgggattgggtcggcggccgctattctctgtggtctgctatcggcttgtccatcgctctg
D. rerio PGI2   tgggactgggtcggtggacgatactccctatggtctgccattggactgtccattgcactt
P. marinus      tgggattgggtgggaggtcgttattcactctggtcggcgatcggcctctccatcgccctg
                *****.***** ** ** ** **.** ** ***** ** **.** .* *****.** **

D. rerio PGI1   cacatcggttttgacaatttcgagcagcttctgtctggtgctcactggatggataatcac
D. rerio PGI2   cacatcggatatgagaactttgagaagctgttggctggagctcattggatggacactcat
P. marinus      cacatcggctttgaaaactttgagcagctgctcagtggagctcactggatg---------
                ******** * *** **.**.*** **** .*   *** *****.******

D. rerio PGI1   ttccgctcggcccctctggagcagaacgctccggtcattctggctctgctcggcgtctgg
D. rerio PGI2   ttccgcacggctcctgtagatcagaacgcacccatgctgctcgcccttttgggaatctgg
P. marinus      ------------------------------------------------------------

D. rerio PGI1   tatgtcaacttcttccaggctgaaacgcatgcactgctgccctacgatcagtacatgcat
D. rerio PGI2   tacatcaacttcttccaggcggagactcactgtctgctgccgtacgatcagtacatgcac
P. marinus      ------------------------------------------------------------

D. rerio PGI1   cgcttcgctgcgtatttccaacagggggacatggagtccaacgggaagtacatcaccaag
D. rerio PGI2   cgcttcgctgcttattttcaacagggtgacatggagtccaatggaaagtacatcacgact
P. marinus      ------------------------------------------------------------

D. rerio PGI1   tctggcactcgtgtgaattaccacacgggacccatcgtttggggagaaccaggaactaat
D. rerio PGI2   aaaggcacgcgggtgaactatcacactggacccatcgtatggggagaaccaggaactaac
P. marinus      ------------------------------------------------------------

D. rerio PGI1   ggacagcacgctttctaccagctcattcaccagggcactcgcttgattccagctgacttc
D. rerio PGI2   ggacagcacgctttctaccagctcatccaccaggggactcgaatggttcctgctgacttc
P. marinus      ------------------------------------------------------------

D. rerio PGI1   ctcattcctgcgcagagccagcatcctatcagagataacctgcatcataagatcctgatg
D. rerio PGI2   ctcattcctgcccaaactcaacatccaatcagaaacagccttcaccacaagattctgttg
P. marinus      ------------------------------------------------------------
```

```
D. rerio PGI1   gcgaatttcctggcgcaaacagaggctctgatgaggggaaagacttctgatgaggctaag
D. rerio PGI2   gcgaattttctggctcaaactgaggcgctgatgaaggggaaaacaacagaagaggcgaag
P. marinus      ------------------------------------------------------------

D. rerio PGI1   aaggagctccaggcttctgggttgtctggagactcgctggagaaactcctgcctcataaa
D. rerio PGI2   aaagagctggaggcgggaggactgagcggagacaacctggagaaaatactaccgcacaaa
P. marinus      ------------------------------------------------------------

D. rerio PGI1   gttttccaaggaaacaagccaagcaactccatcatctttaagaaacttacacccttcatg
D. rerio PGI2   gttttccagggaaacaagccaacaaactccatcgtcttcaagaagctctctccgttcact
P. marinus      ------------------------------------------------------------

D. rerio PGI1   cttggtgcactggttgcgatgtatgagcacaagatcttcgtgcagggtgtgatgtggaat
D. rerio PGI2   ctgggtgtgctgatcgccatgtatgaacacaagatcttcattcagggcgtcatgtgggaa
P. marinus      ------------------------------------------------------------

D. rerio PGI1   atcaacagctatgatcagtggggcgtcgaactcggcaagcaactggccaagaagatcgaa
D. rerio PGI2   atcaacagctttgatcagtggggggttgagctgggtaaacagctggcgaaaaagatcgag
P. marinus      ------------------------------------------------------------

D. rerio PGI1   cccgagctgcaggacgatgcggaggttcattcccacgactcctccaccaatggactcatt
D. rerio PGI2   ccggagctccaggattcagctcaggtcagctctcatgattcctccaccaacggcctcatc
P. marinus      ------------------------------------------------------------

D. rerio PGI1   ggattcttcaagaagaaccgcttttag
D. rerio PGI2   aacttcctcaaaaacaactttgcttga
P. marinus      ---------------------------
```
